# Supplementary material for: Systematic detection of co-infection and intra-host recombination in more than 2 million global SARS-CoV-2 samples
Source: Nat Commun. 2024 Jan 15;15:517. doi: 10.1038/s41467-023-43391-z (PMC10789779; doi:10.1038/s41467-023-43391-z)
Supplement: Supplementary file 1 — Supplementary Information [file 41467_2023_43391_MOESM1_ESM.pdf]

Supplementary Information

Systematic detection of co-infection and intra-host recombination in more than 2 million global SARS-CoV-2 samples

Orsolya Anna Pipek, Anna Medgyes-Horváth, József Stéger, Krisztián Papp, Dávid Visontai, Marion Koopmans, David Nieuwenhuijse, Bas B. Oude Munnink, VEO Technical Working Group, István Csabai

Contents:

Supplementary Figures 1-7.

Supplementary Methods 1-3.

Supplementary References

Supplementary Figure 1. The number of samples in the CoVEO database. **a.** The number of good-quality SARS-CoV-2 samples with a human host assigned to different variants in the CoVEO database. **b.** The relationship between the total number of samples assigned to a specific variant and the number of coinfection samples containing the variant. The straight black line represents a linear dependence with a slope of 1 on a log-log graph. R represents the Pearson-correlation coefficient, and the corresponding p-value is derived from a two-sided t-test (n=13 variants). A higher resolution version of the figure can be found at https://github.com/csabaiBio/SARSCoV2-coinf/blob/main/SuppFigures/SuppFig1.pdf

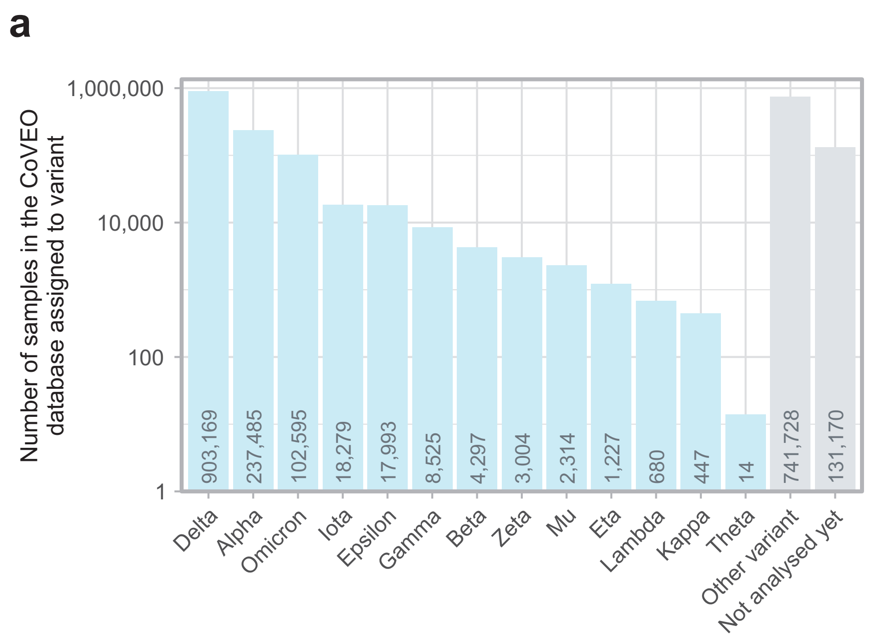

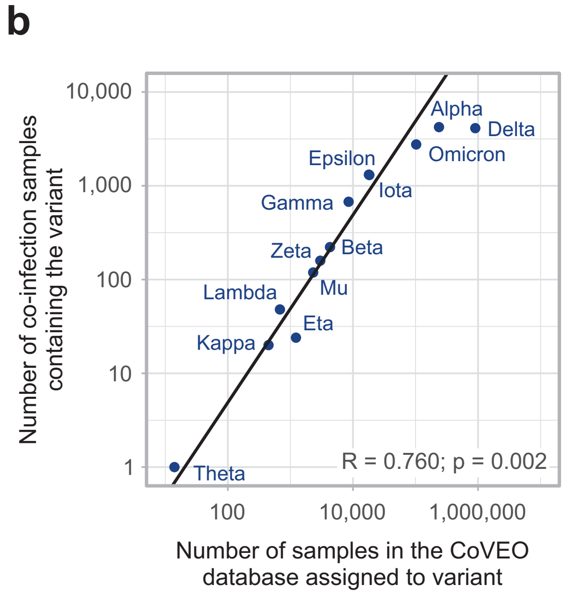

Supplementary Figure 2. Temporal distribution of co-infection samples for variant combinations with more than 50 samples. (The same figures for the top 4 most abundant combinations are shown in Figure 1c of the main manuscript.) Prevalence curves indicate the number of GISAID[1] samples assigned to the respective variants (binned weekly). Blue vertical lines on the bottom panels mark the collection date of co-infection samples of the given variants. A higher resolution version of the figure can be found at https://github.com/csabaiBio/SARSCoV2-coinf/blob/main/SuppFigures/SuppFig2.pdf

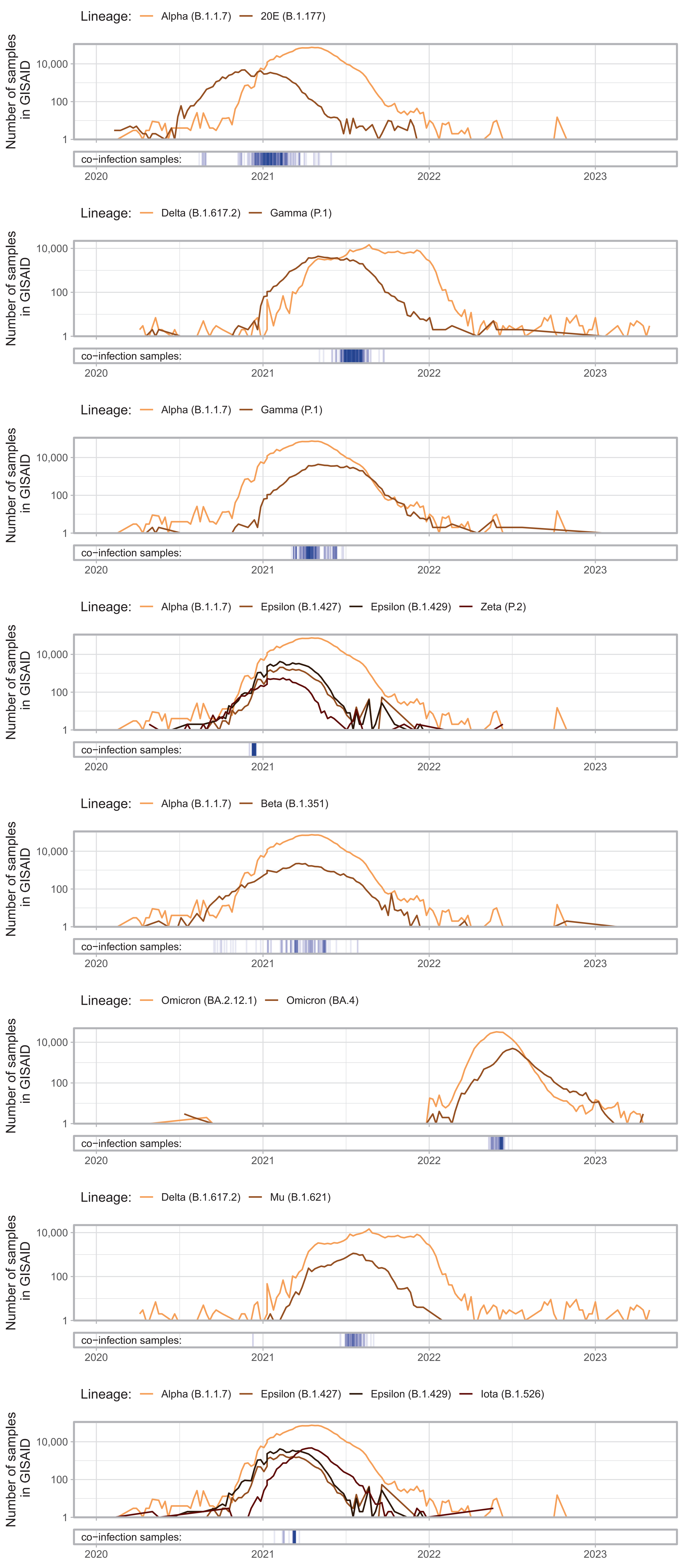

Supplementary Figure 3. Temporal distribution of co-infection samples for countries with at least 1,000 good-quality samples in the database. The top panel on each subfigure depicts the number of samples in the CoVEO database collected each week from the appropriate country. The second panel shows the collection date of co-infection samples identified in the given country. The third panel demonstrates the country-wise weekly prevalence curves of variants in the GISAID database[1] that were included in the study. Coloured curves correspond to the most abundant variants in the whole set of detected co-infection samples (Delta, Omicron (BA.1), Alpha, Iota, Epsilon, 20E), while different shades of grey demonstrate the remaining lineages. For the United States and the United Kingdom, on the fourth panel various measures (the number of concurrently circulating lineages, the cumulative number of lineages and information entropy) of genetic diversity are plotted based on GISAID data[1] from the given country. A higher resolution version of the figure can be found at https://github.com/csabaiBio/SARSCoV2-coinf/blob/main/SuppFigures/SuppFig3.pdf

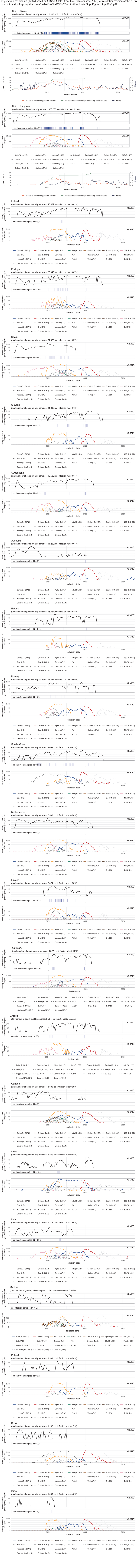

Supplementary Figure 4. Weekly co-infection rate in the function of genetic diversity. Weekly coinfection rate was calculated as the percentage of co-infection samples out of all good-quality samples in the given country, in the given week. Genetic diversity was either defined as the number of linages concurrently present in the given country in the given week in GISAID data[1] (left panels) or the information entropy (right panels, see Methods). Only lineages investigated by the present study were considered. Weeks for which the number of good-quality samples in the CoVEO database did not reach 10 were discarded. Each marker represents the data for a single week. Marker size corresponds to the number of good-quality samples available. "R" and "p" indicate Pearson-correlation coefficients and respective two-sided t-test p-values (non-significant ($p \geq 0.05$) results are not displayed). A higher resolution version of the figure can be found at https://github.com/csabaiBio/SARSCoV2-coinf/blob/main/SuppFigures/ SuppFig4.pdf

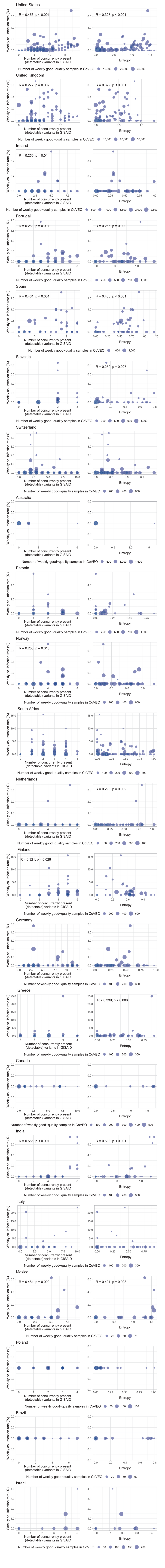

Supplementary Figure 5. Mutually exclusive defining mutations in GISAID[1] samples assigned to the XD, XF and XS 'Deltacron' (recombinant Delta and Omicron) lineages. Samples (rows) are listed by their GISAID ID, with their Pango lineage[2] indicated in brackets. Defining mutations (columns) are coloured by their respective variant (blue for Delta and red for Omicron). Heatmap colours show whether the given sample contains the given mutation or not (dark blue: present Delta-mutation; light blue: missing Omicron-mutation; dark red: present Omicron-mutation; light red: missing Delta-mutation). Dashed vertical lines mark the recombination breakpoint range(s) for each lineage. (The same genomic regions are indicated with red shaded areas in Figure 4 of the main manuscript.) A higher resolution version of the figure can be found at https://github.com/csabaiBio/ SARSCoV2-coinf/blob/main/SuppFigures/SuppFig5.pdf

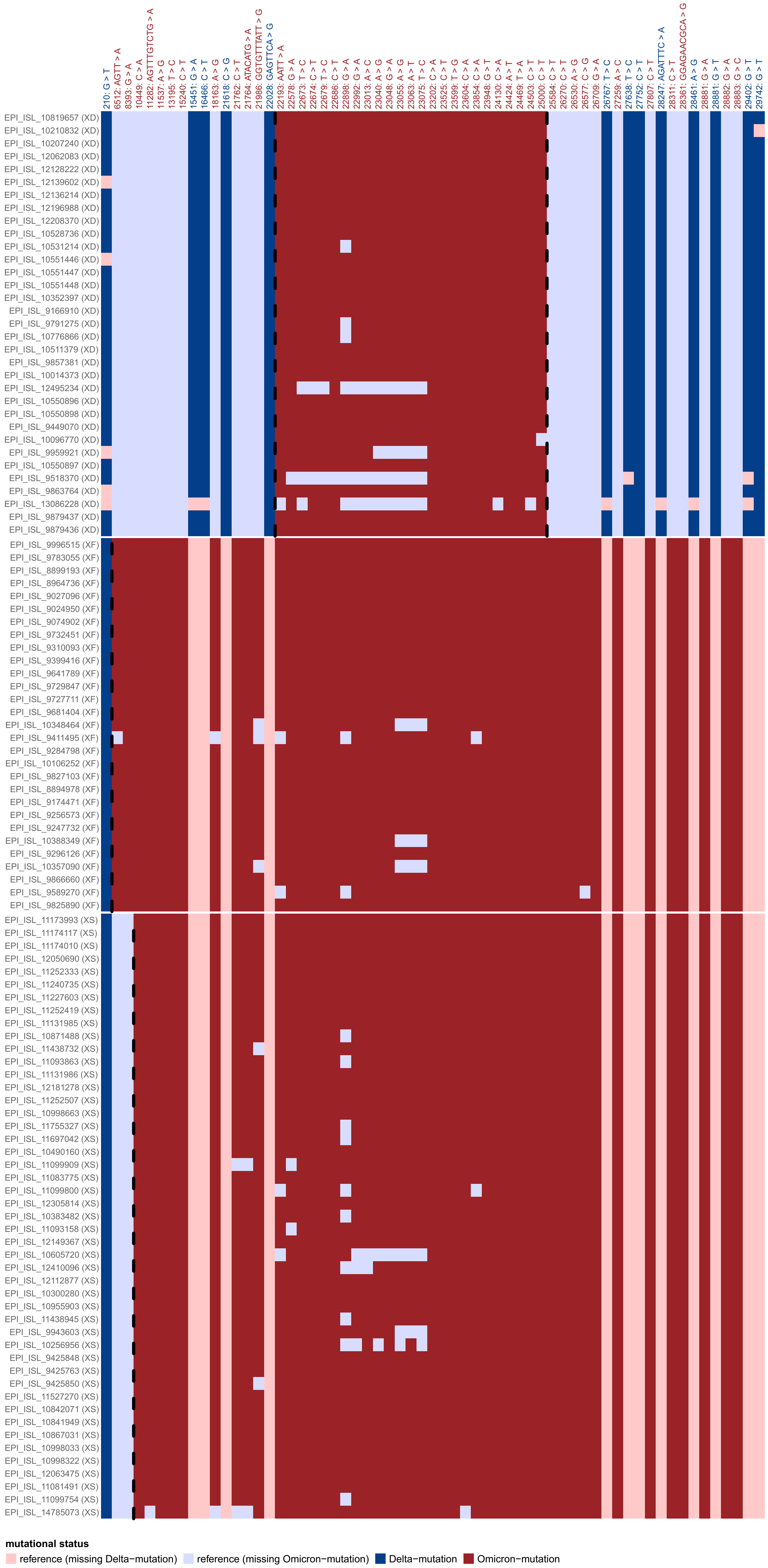

Supplementary Figure 6. Distribution of sequencing platforms and instruments within all good-quality samples included in the study, for co-infection samples and for samples selected for downstream read-level analysis. A higher resolution version of the figure can be found at https://github.com/csabaiBio/SARSCoV2-coinf/blob/main/SuppFigures/SuppFig6.pdf

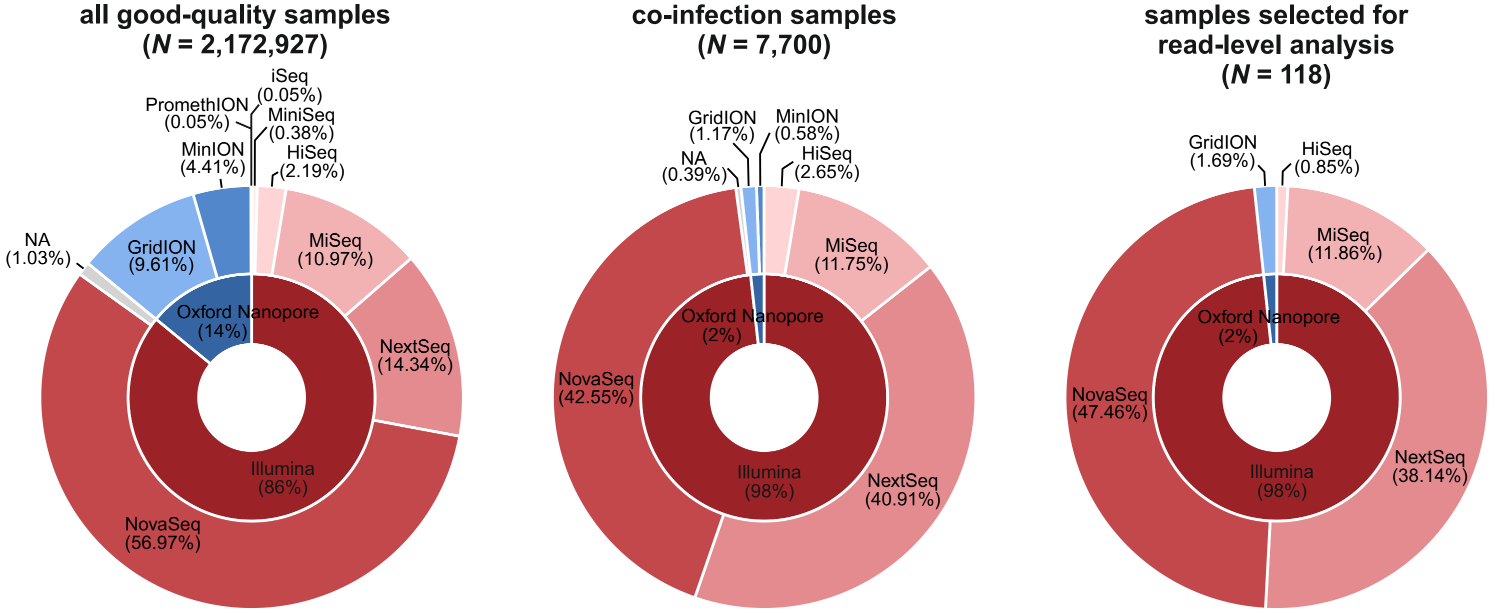

Supplementary Figure 7. Schematic diagram of the workflow used to produce the data analysed in the study. Only those steps of the VEO[3] variant calling pipeline[4,5] and features of the COVID-19 Data Portal[6] are shown that are relevant to the current analysis. More details on both can be found in Rahman et al.[7]. For the CoVEO database, only those tables and fields are displayed that were queried during data processing. Queries, codes, data files and visualizations are uploaded to the csabaiBio/SARSCoV2-coinf github repository. Postgres, PostgreSQL and the Slonik Logo are trademarks or registered trademarks of the PostgreSQL Community Association of Canada and used with their permission. A higher resolution version of the figure can be found at https://github.com/csabaiBio/SARSCoV2-coinf/blob/main/SuppFigures/SuppFig7.pdf

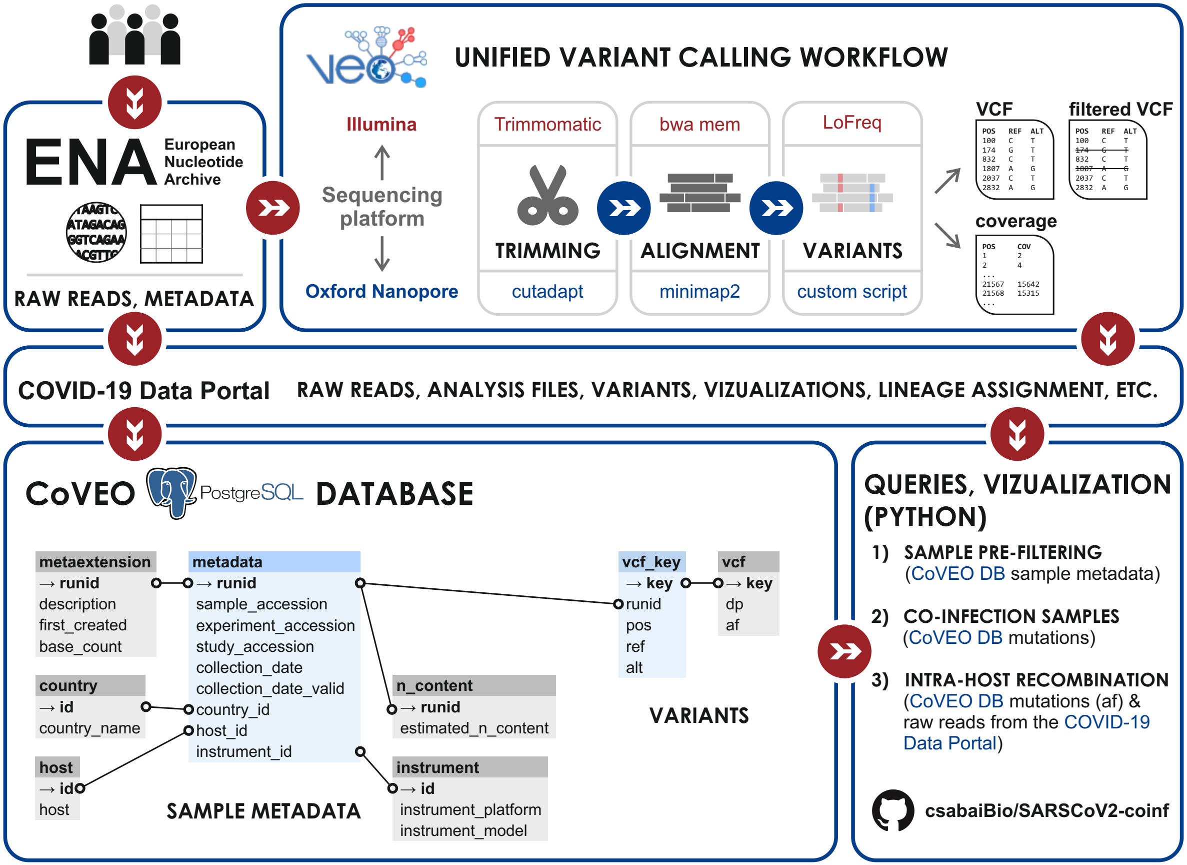

Supplementary Method 1. - analysis pipeline

PostgreSQL queries for co-infection detection using the CoVEO database

The online HTML version of the notebook contains codes for straightforward reproducibility and can be downloaded from https://github.com/csabaiBio/SARSCoV2-coinf/blob/main/pipelines/SuppFile1__coinf__pipeline.html. In this notebook, we query the CoVEO PostgreSQL database to identify SARS-CoV-2 co-infection samples. To this end, we select samples that carry a convincing ratio of unique defining mutations of multiple variant strains. We further refine the set of samples by considering all mutually exclusive defining mutations of their variant combination, finally setting a threshold of 80% for the ratio of mutually exclusive defining mutations that have to be present in all composing variants for a sample to be deemed a co-infection case.

Table of contents

1 Input and output files **2**

2 The CoVEO database **2**

3 Unique defining mutations of SARS-CoV-2 variants **3**

4 Collecting candidate co-infection samples **4**

4.1 Quality filtering 4

4.2 Filtering for unique defining mutations 4

5 Refining candidates with mutually exclusive defining mutations **4**

6 Final selection of co-infection samples **5**

7 Variant compositions of co-infection samples **6**

8 Distribution of variants in the database **6**

8.1 Number of good quality samples for each variant 6

8.2 Correlation between number of samples and co-infection samples 6

9 Collecting metadata for co-infection samples **8**

10 Study-specific prevalence of co-infection samples **8**

11 Country-specific prevalence of co-infection samples **8**

1 Input and output files

- Data files needed as input:
  - list of mutations with lineage-specific prevalences in the GISAID database
    - datatable provided by github.com/rvalieris/LCS
    - original paper describing the data by Valieris et al.[8]
- Data files generated as output:
  - Supplementary Data 1-4. (https://github.com/csabaiBio/SARSCoV2-coinf/tree/main/SuppTables)
  - Additional data files 1-4. (https://github.com/csabaiBio/SARSCoV2-coinf/tree/main/data)

2 The CoVEO database

The CoVEO database is a PostgreSQL database storing the mutational data (VCF files) of SARS-CoV-2 sequencing samples uploaded to the European COVID-19 Data Portal, maintained partly by the efforts of the Versatile Emerging infectious disease Observatory (VEO) consortium. The dataset is unique in the sense that besides the commonly available consensus sequences of the samples, it also contains low alternate allele-frequency mutations and sequencing depth information in a straightforwardly queryable format, along with sample metadata to allow for simple filtering. Additionally, samples of the database are analysed with a standardized variant calling workflow (available on GitHub) in order to keep technical bioinformatics artefacts at a minimum and to obtain comparable results in spite of multiple sample collectors and various laboratory protocols.

! Important

This notebook uses PostgreSQL queries and python code to collect and further analyse data. Upon reasonable request, we provide access to the CoVEO database. Please, e-mail **kooplex@elte.hu** with any inquiries.

Date of latest modification in the database:

	max
0	2023-05-19 00:40:13.801316

Total number of samples with a human host (with appropriate metadata) in the database:

	total_number_of_human_samples
0	3093454

Total number of *good quality* samples with a human host (with appropriate metadata) in the database:

	total_number_of_goodq_samples
0	2172927

Time range for the collection date of good quality samples:

	earliest	latest
0	2019-12-30	2022-06-30

3 Unique defining mutations of SARS-CoV-2 variants

Instead of using a precompiled list of genomic variations characteristic of each SARS-CoV-2 viral strain, we used the marker table provided by Valieris et al. in which all distinguishing mutations are listed with the number of GISAID samples containing the reference and alternate alleles for each lineage.

As an initial step, we selected the mutations listed in this table that were unique and highly indicative of specific viral strains. More precisely, for each mutation, the lineages with the largest and second-largest prevalence were identified. Genomic variations with a largest prevalence of larger than 80% and a second-largest prevalence of less than 10% were considered as "unique defining mutations" of the lineage with the highest mutational incidence. The number of unique defining mutations of each strain are listed below.

> **Note**
>
> The following code generates **Supplementary Data 2.** and **Supplementary Data 3.**

Table 5: Number of unique defining mutations in different lineages

Variant	Number of unique defining mutations
Mu_B.1.621	22
Gamma_P.1	20
Eta_B.1.525	19
Alpha_B.1.1.7	19
AV.1	18
A.23.1	17
Theta_P.3	16
B.1.1.318	15
Omicron_BA.1	14
Lambda_C.37	13
B.1.623	10
Beta_B.1.351	8
B.1.617.3	8
B.1.177	6
Zeta_P.2	6
Kappa_B.1.617.1	5
Delta_B.1.617.2	5
Iota_B.1.526	4
Epsilon_B.1.427_429	4
Omicron_BA.2.12.1	4
Omicron_BA.5	3
Omicron_BA.3	2
Omicron_BA.4	2

4 Collecting candidate co-infection samples

4.1 Quality filtering

The altogether 3,093,454 samples of a human host in the CoVEO database were initially filtered to exclude samples that had a total base count of 100,000 or less to avoid misinterpreting sparse sequencing data. Additionally, to further ensure relatively even coverage of the viral genome, we discarded samples that had a sequencing depth of less than 10 in more than 10% of the 29,903 genomic positions of the reference genome (NCBI ID: NC_045512.2). This filtering step resulted in 2,172,927 remaining samples.

4.2 Filtering for unique defining mutations

Good-quality samples were considered to be putative co-infection samples if

- at least 50% of unique defining mutations
- of at least two different variants

were present in them. Mutations were not filtered for allele frequency.

> **ℹ Note**
>
> The following code generates **Additional Datafile 1.**

```
Number of candidate co-infection samples: 29666
Number of unique variant combinations: 1270
```

5 Refining candidates with mutually exclusive defining mutations

Given that the number of defining mutations in a given variant greatly affects detectability of co-infection cases, we re-evaluated each sample from the above list of putative co-infection samples based on the following procedure:

1. For the variant composition of the given sample, we identified "mutually exclusive defining mutations" of the comprising variants. We expanded the list of unique markers to all mutations that had a largest prevalence of at least 80% in GISAID samples in one of the variants of the given composition, but a second-largest prevalence of no more than 10% in all other variants *of the given variant composition.*
2. If the candidate sample had less than 50% of the *mutually exclusive* defining mutations of *any* of its variants, it was discarded from further analysis.

> **ℹ Note**
>
> The following code generates **Additional Datafile 2.** and **Supplementary Data 4.**

```
Number of refined candidate co-infection samples: 22180
Number of unique variant combinations for refined samples: 711
```

6 Final selection of co-infection samples

The number of supposed co-infection samples was then determined with multiple thresholds for the ratio of required mutually exclusive defining mutations in the range of 0.5 to 1, and the final filtering limit of 0.8 was chosen for the identification of a total number of 7,700 co-infection samples.

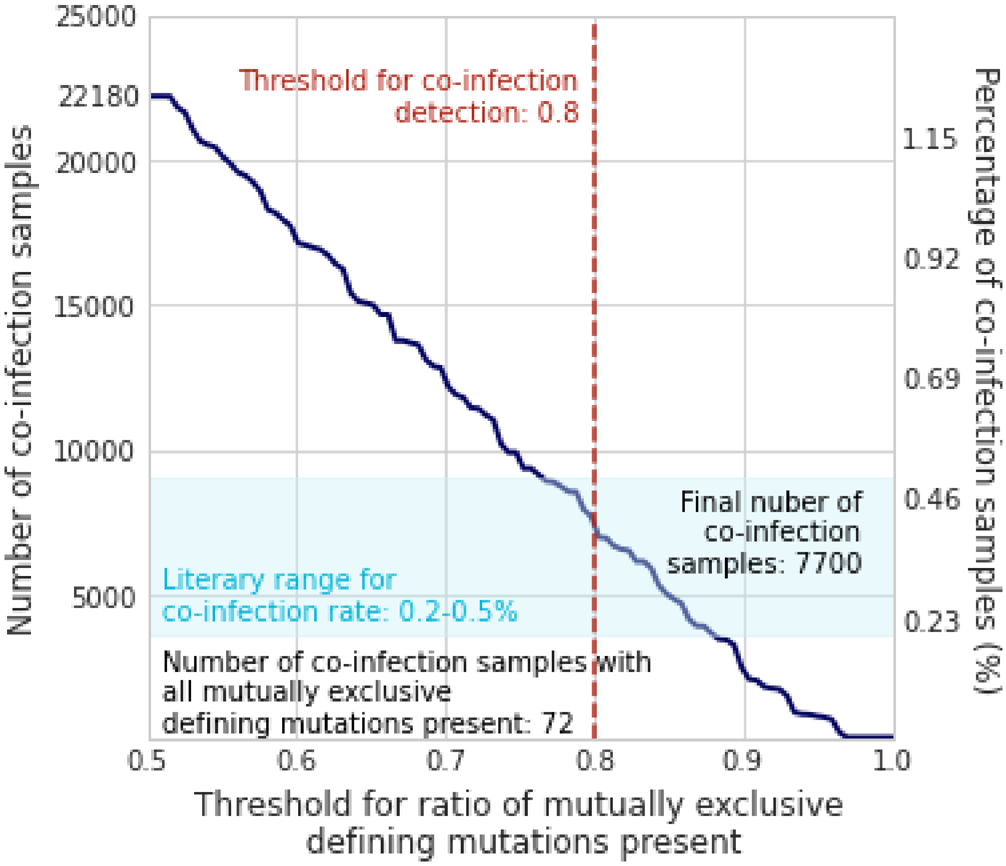

Figure 1: The number and percentage of co-infection samples identified in the CoVEO database with different thresholds for the required ratio of mutually exclusive defining mutations present in the variants. The literary range for co-infection rate is indicated with the blue rectangle. The chosen threshold of the ratio of defining mutations for co-infection detection is marked with the vertical red, dashed line, corresponding to the value of 0.8. The number of co-infection samples in which all mutually exclusive defining mutations of all comprising variants were present was 76.

This corresponds to a co-infection rate of:

```
0.35%
```

7 Variant compositions of co-infection samples

> **Note**
>
> The following code generates **Additional Datafile 3.**

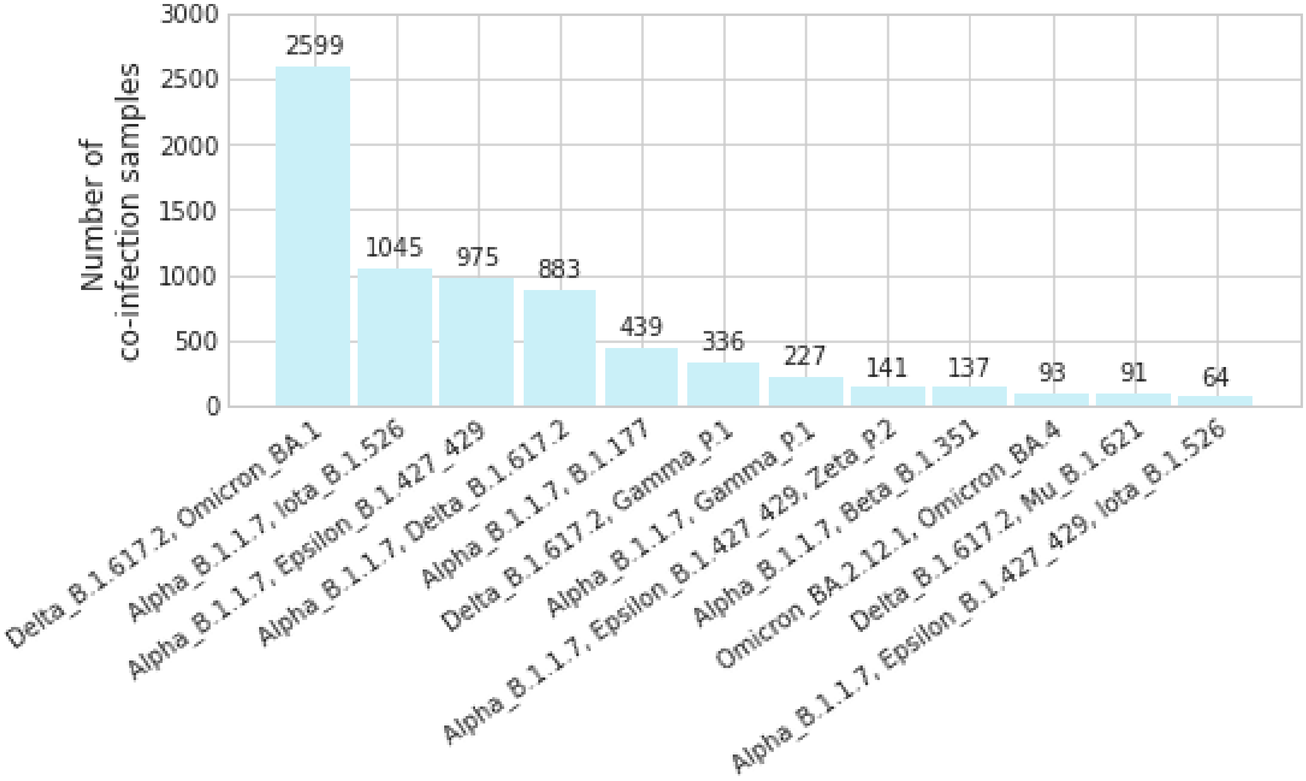

Figure 2: The number of co-infection samples detected with different variant compositions. Variant compositions with less than 50 co-infection samples are not shown.

8 Distribution of variants in the database

8.1 Number of good quality samples for each variant

8.2 Correlation between number of samples and co-infection samples

The figure below shows the number of co-infection samples that include the given variant in function of the number of good quality samples assigned to the given variant in the database. (Note the logarithmic axes.) It is apparent that the more samples are available from a given variant, the more likely it is to detect co-infection samples that include that variant in their variant composition. The grey line represents a linear relationship between the two values, i.e. a straight line with a slope of 1 in a log-log plot.

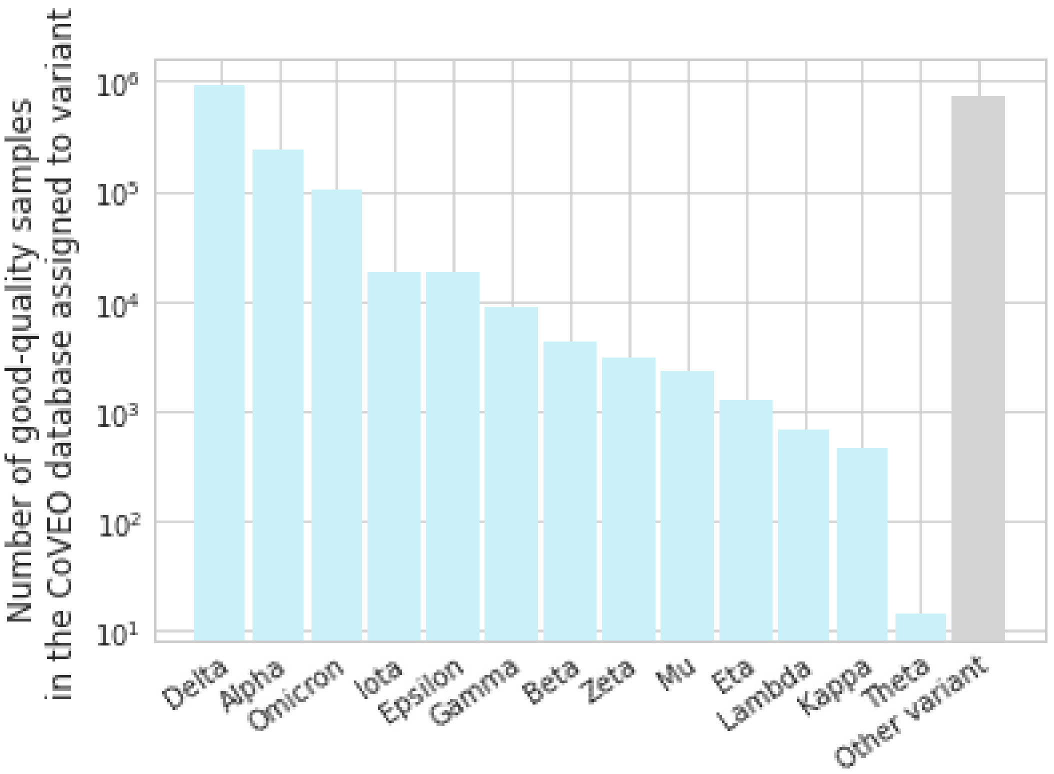

Figure 3: The number of all good-quality samples in the CoVEO database assigned to specific variants.

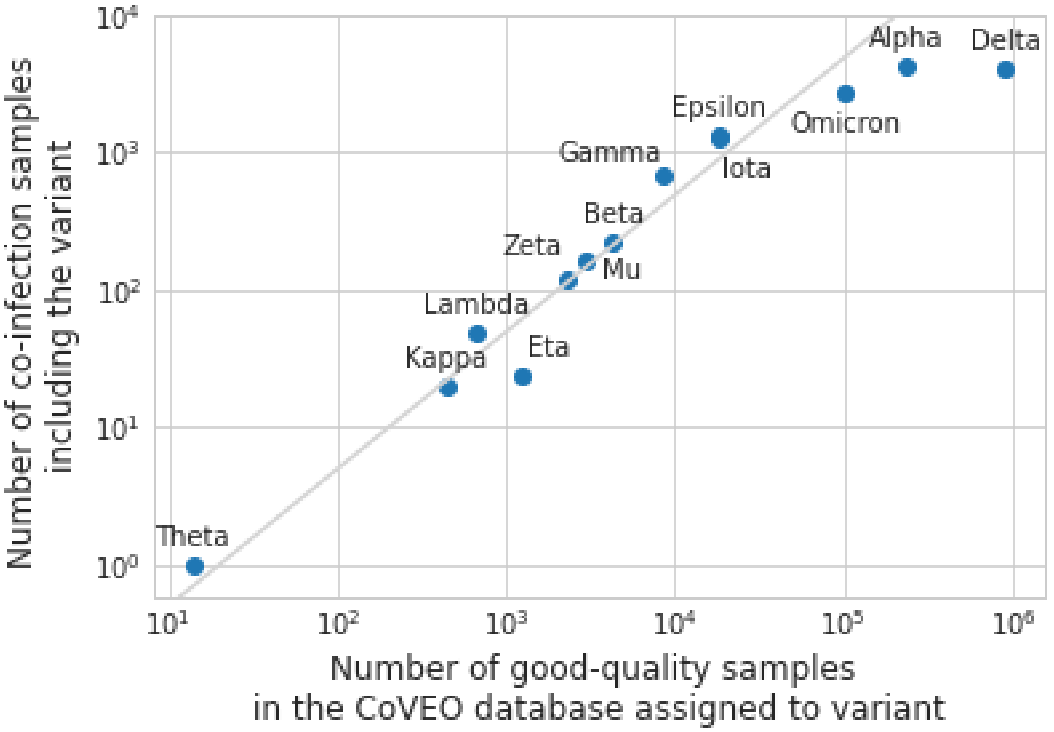

Figure 4: Correlation between the total number of good-quality samples assigned to specific variants and the number of co-infection samples with the given variant in their variant composition.

9 Collecting metadata for co-infection samples

Once the set of co-infection samples have been identified, we further query the database to collect their available metadata.

> **Note**
>
> The following code generates **Additional Datafile 4.**

10 Study-specific prevalence of co-infection samples

To see how co-infection samples are distributed across different studies, we determined the number of good-quality samples of a human host with available mutation information of various study accession IDs in the CoVEO database. Study-specific prevalence rates were calculated as the percentage of identified co-infection samples within a given study.

> **Note**
>
> The following code generates **Supplementary Data 1.**

Table 6: Studies with a co-infection prevalence of at least 10%

ENA study accession	Number of co-infection samples	Number of good quality samples in the study	Co-infection prevalence (%)
PRJNA817870	117	152	76.9737
PRJNA817806	44	101	43.5644
PRJNA853723	82	97	84.5361
PRJNA809680	5	8	62.5
PRJNA827817	5	7	71.4286
PRJNA748832	1	6	16.6667
PRJNA698337	1	6	16.6667
PRJNA728440	1	4	25
PRJNA804575	1	1	100

The listed studies either contain very few samples or were specifically pre-selected to include large amounts of co-infection cases (studies PRJNA817870, PRJNA827817, PRJNA853723, PRJNA817806, PRJNA809680).

11 Country-specific prevalence of co-infection samples

To see how co-infection samples are distributed across different countries, we determined the number of good-quality samples of a human host with available mutation information of various countries in the CoVEO database. Country-specific prevalence rates were calculated as the percentage of identified co-infection samples within a given country.

Supplementary Method 2. - analysis pipeline

Allele frequency-based identification of putative co-infection samples containing trace amounts of recombinant genomes

The online HTML version of the notebook contains codes for straightforward reproducibility and can be downloaded from https://github.com/csabaiBio/SARSCoV2-coinf/blob/main/pipelines/SuppFile2__AF__pipeline.html. In this notebook, we investigate the alternate allele frequency distributions measured in co-infection samples at the genomic positions of mutually exclusive variant-defining mutations. Our analysis reveals a systematic bias in standardized allele frequency values in samples of specific variant combinations. To detect putative recombinants based on alternate allele frequency shifts along the genome, we employ an initial hard-filtering step on coinfection samples and finally refine our results by introducing a pipeline which is aimed to correct for the above-described bias in alternate allele frequency distributions in specific genomic positions. Finally, a list of putative intra-host recombinant samples is selected.

Table of contents

1 Input and output files 1

2 Alternate allele frequency distributions at defining mutations 2
- 2.1 Standardized alternate allele frequencies . . . 2
- 2.2 Possible causes of bias in alternate AFs . . . 4
- 2.3 Estimation of standardized AF distributions . . . 4

3 Identification of putative recombinants 6
- 3.1 Hard-filtering of co-infection samples . . . 6
- 3.2 Correction for AF-distribution bias . . . 7

1 Input and output files

- Data files needed as input:
  - Additional data file 3 (https://github.com/csabaiBio/SARSCoV2-coinf/blob/main/data/datafile3.tar.gz)
  - Additional data file 4 (https://github.com/csabaiBio/SARSCoV2-coinf/blob/main/data/datafile4.tar.gz)

2 Alternate allele frequency distributions at defining mutations

2.1 Standardized alternate allele frequencies

A naive hypothesis would suggest that the alternate allele frequencies (AFs) measured at mutually exclusive defining mutations in a co-infection sample should directly reflect the variant proportions comprising the sample, i.e. a Delta – Omicron (BA.1) sample with variant ratios 80%-20% (respectively) should have alternate AFs at mutually exclusive Delta-defining mutations of around 0.8 and at mutually exclusive Omicron (BA.1)-defining mutations of around 0.2. However, literary evidence (Bal et al., 2022) proves that this is usually not the case, mostly due to systematic bias introduced by the primers used for sequencing.

Here we investigate the alternate AF distribution in co-infection samples of specific variant compositions. To get comparable results across samples, alternate AFs measured at mutually exclusive variant-defining positions are standardized with the mean and standard deviation of AFs within the given sample for the given variant, so that the mean of standardized values is 0, while their standard deviation is 1. Thus in a sample of variant composition $A - B$, the $\overline{AF}_{s,p,A}$ standardized AF in a mutually exclusive defining mutation of variant A, at genomic position g is:

$$\overline{AF}_{s,g,A} = \frac{AF_{s,g,A} - \mu_{s,A}}{\sigma_{s,A}}$$

where $\mu_{s,A}$ is the mean and $\sigma_{s,A}$ is the standard deviation of alternate AFs measured in defining positions of variant A, in sample s.

The figures below show the standardized AFs in samples of the top 4 most frequent variant compositions separately for the mutually exclusive defining mutations of the comprising variants.

ℹ Note

Out of the 7,700 previously identified co-infection samples, only those 7,290 are considered here that had exactly two comprising variant strains.

a)

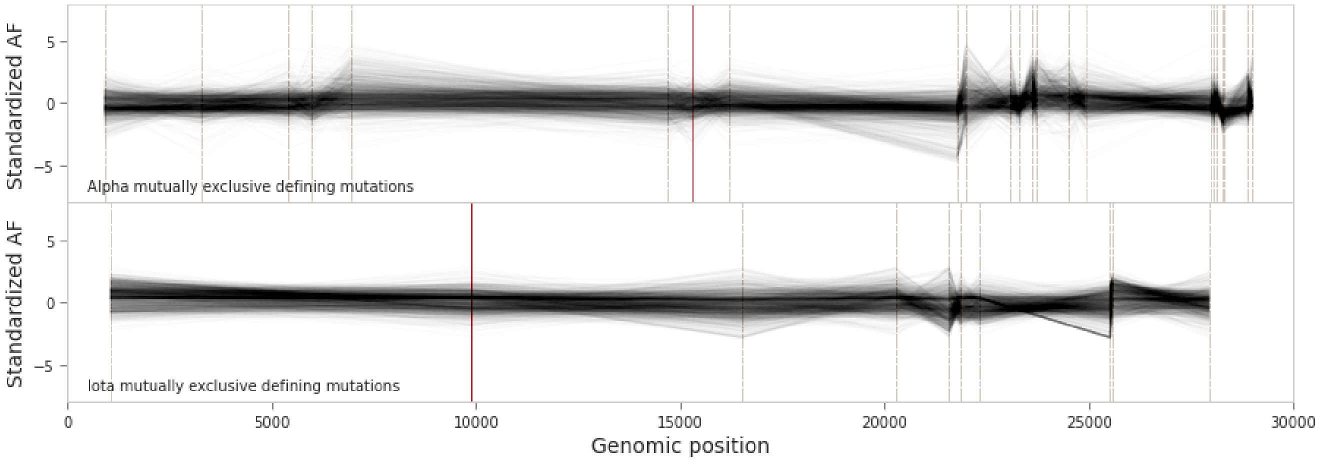

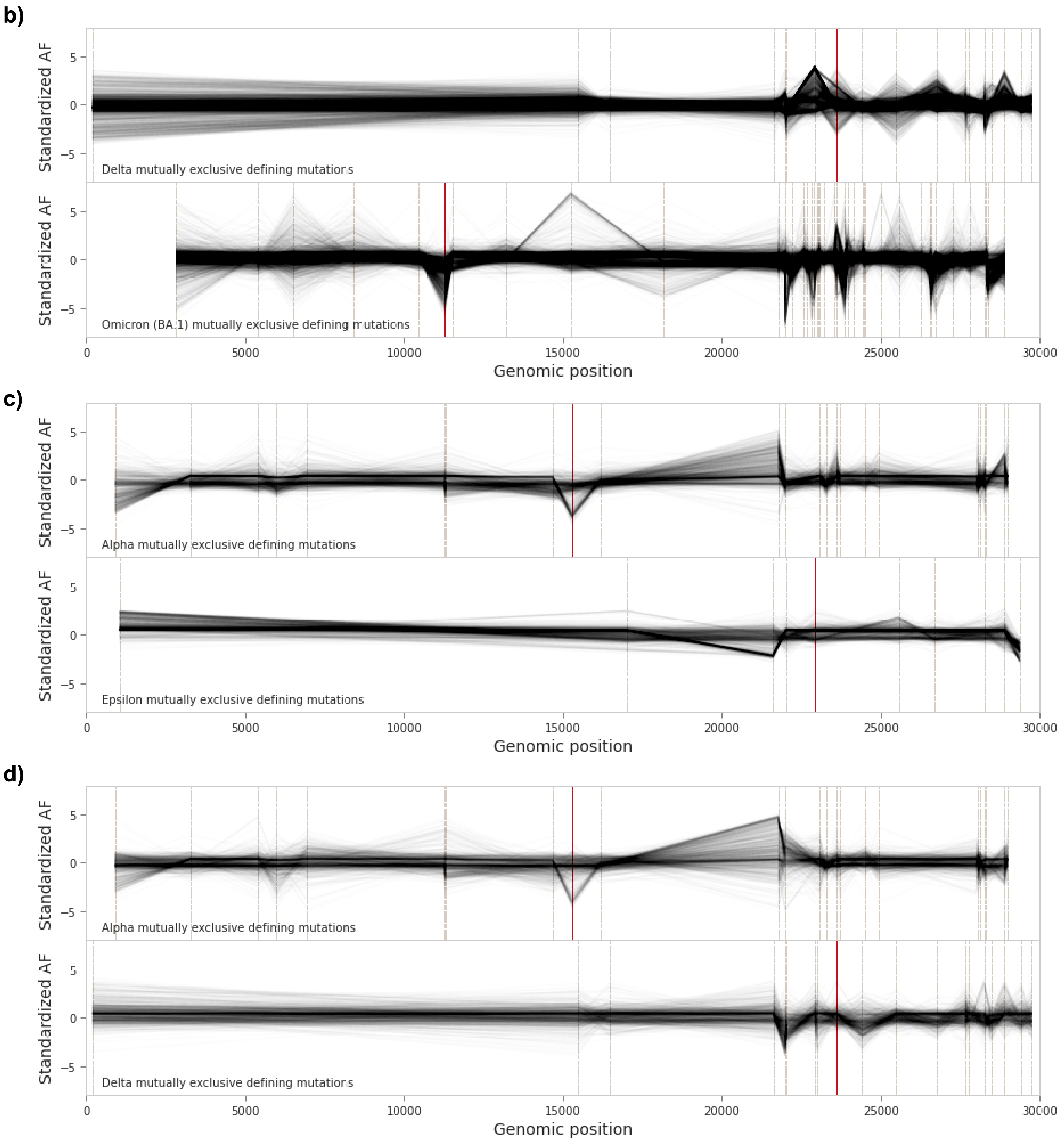

Figure 1: Standardized alternate AF distribution in samples of the most frequent variant combinations. Vertical dashed lines mark the genomic positions of mutually exclusive defining mutations of the given variant in terms of the specific variant composition. Vertical red lines indicate positions singled out for demonstrative purposes below. a) Alpha – Iota co-infection samples. b) Delta – Omicron (BA.1) co-infection samples. c) Alpha – Epsilon co-infection samples. d) Alpha – Delta co-infection samples.

2.2 Possible causes of bias in alternate AFs

It is apparent from Fig. 1 that in some genomic positions, standardized AF values tend to markedly differ from zero. We hypothesize that this effect might due to the preferential attachment of primer sequences to genomes containing or lacking specific mutations.

It is also evident that these systematic biases vary based on the specific variant composition under investigation. For example, standardized alternate AFs at the Alpha-defining synonymous 15279:C>T mutation are around zero in Alpha – Iota samples (Fig. 1a), while they can have much lower values in Alpha – Epsilon (Fig. 1c)) and Alpha – Delta (Fig. 1d)) samples. This observation also supports the theory that the primers used during PCR amplification can have a preference for specific genome sequences, which is directly influenced by the set of available genomes (i.e. the variant composition).

To check this assumption, we selected Delta – Omicron (BA.1) co-infection samples for further analysis. We binned the genome into regions of 500 bp and selected those that overlapped at least one Delta- and one Omicron (BA.1)-defining mutation. In these regions, we calculated the mean value of standardized alternate AFs of all mutually exclusive defining mutations that overlapped the given region, separately for Delta- and Omicron (BA.1)-defining mutations and for each sample. These mean values are plotted in Fig. 2a and Fig. 2b, for Delta- and Omicron (BA.1)-defining positions, respectively. In Fig. 2c, these values are displayed simultaneously on the same chart and in Fig. 2d, the sum of these values is shown for each region, for each sample. In regions indicated by the vertical arrows, the mean standardized AFs of Delta- and Omicron (BA.1)-defining mutations are shifted from zero in the opposite direction and their sum shows a narrower distribution around zero. This suggests that in these regions, genomes of one of the two comprising variants are disproportionately amplified, while the relative abundance of the other variant is consequently lowered.

2.3 Estimation of standardized AF distributions

Given that our main approach for detecting traces of recombination in co-infection samples is to identify putative breakpoints based on alternate AF shifts along the genome, it is important to distinguish between true signals and ones simply caused by the above detailed inherent variation in AF values. To this end, we estimate the standardized alternate AF distribution at each mutually exclusive defining mutation of each variant for each variant composition separately. In other words, we fit a bimodal probability density distribution to the normalized histogram of the data points at each vertical line in Fig. 1, and do the same for all additionally relevant variant compositions.

The generic form of the function we use for fitting is the following:

```
def bimodal(x, mu1, s1, mu2, s2):
    f = (1/(s1*np.sqrt(2*np.pi)))*np.exp((-1/2)*((x-mu1)/s1)**2)
    f += (1/(s2*np.sqrt(2*np.pi)))*np.exp((-1/2)*((x-mu2)/s2)**2)
    return f/2
```

The following figures show the results of the fitting procedure for the genomic positions indicated by red vertical lines in Fig. 1.

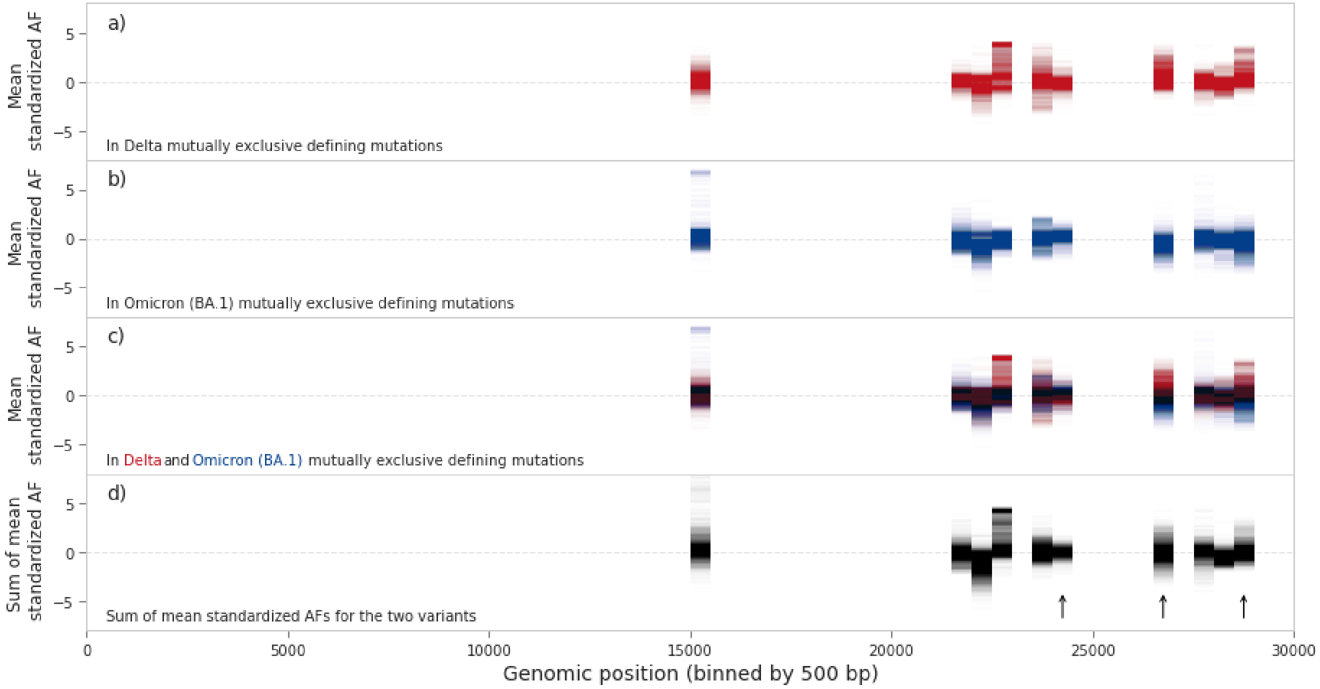

Figure 2: Regional means of standardized alternate AFs of mutually exclusive variant-defining mutations in Delta – Omicron (BA.1) samples, calculated separately for each sample. a) Regional means of standardized alternate AFs of mutually exclusive Delta-defining mutations. b) Regional means of standardized alternate AFs of mutually exclusive Omicron (BA.1)-defining mutations. c) The previous values plotted together. d) Sum of regional means of standardized alternate AFs of mutually exclusive variant defining mutations. Arrows indicate regions where the preferential amplification of one of the variants is compensated by the lowered relative abundance of the other.

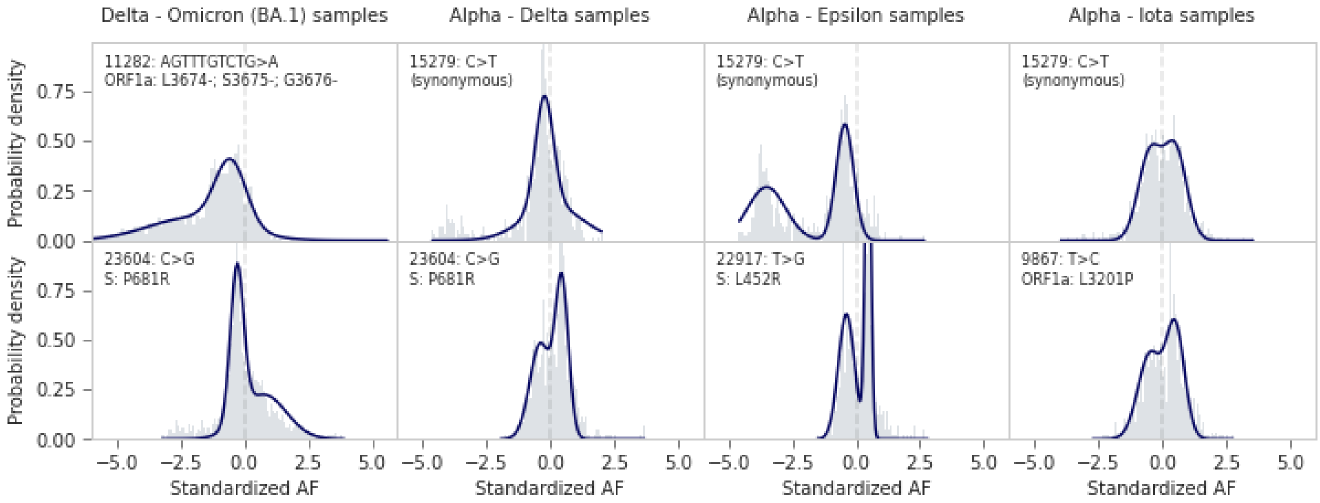

Figure 3: Standardized alternate AF distributions in specific variant defining mutations in samples of different variant combinations. These genomic positions correspond to the ones indicated by red vertical lines in Fig. 1. Grey bars show the normalized histogram of measured values, while blue lines depict the fitted bimodal distribution.

3 Identification of putative recombinants

3.1 Hard-filtering of co-infection samples

Clonally recombinant samples would in principle have genomes that were fused together from the appropriate parts of the genomes of parental viral strains at some breakpoint(s), thus would exhibit signs of different sets of mutually exclusive defining mutations of their parental variants before and after the breakpoint(s). In practice, however, the two parental strains usually co-exist with the recombinant strain (with varying ratios) within a single sample. In theory, assuming no bias in AF distributions, putative recombinant breakpoints could be identified from the shifts in AFs observed for the variant-defining mutations of the parents. In an ideal setting, the absolute value of the AF shift corresponds to the ratio of the recombinant genome in the sample (Fig. 4).

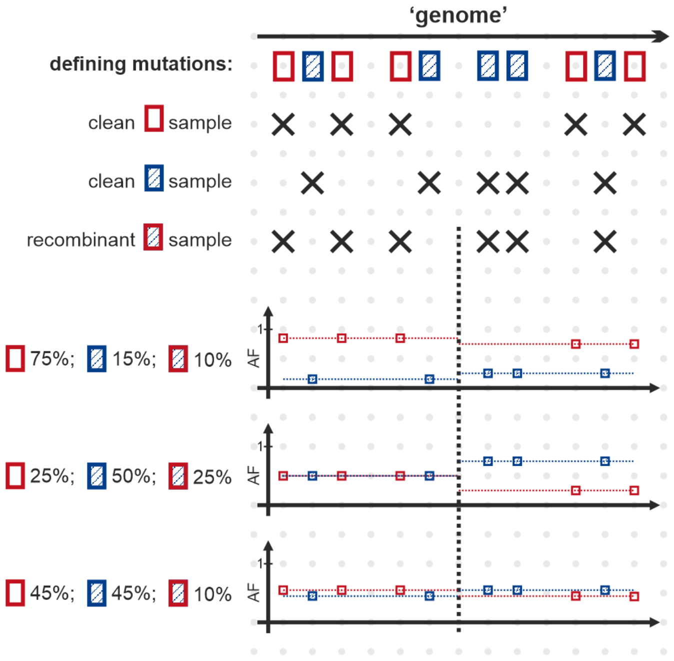

Figure 4: Theoretical AF shifts of defining mutations occurring at a recombinant breakpoint in samples containing the mixture of two parental and a recombinant strain with different ratios.

To select samples with supposed evidence of the presence of recombinant genomes, we developed a pipeline that detects putative breakpoints in co-infection samples where the mean alternate AF of one set of mutually exclusive defining mutations increases, while the mean alternate AF of the other set of mutually exclusive defining mutations decreases. To filter out presumed artefacts and noise, only those genomic positions were retained as possible breakpoints

- where the absolute AF shift for both variants was 0.05 or larger,
- that separated the genome in a way that resulted in at least four variant-defining mutations of both parental strains on each side of the position,
- where the absolute AF shift for both variants was larger than the standard deviation of AFs on either side of the position,
- which resulted in genomic segments where the standard deviations of AFs in the defining mutations of either variant were lower than 0.1.

Note

In this analysis (as well as previously), all mutually exclusive lineage-defining mutations were considered for the given variant combination to increase statistical power.

```
Starting processing...
>>>>>>>>>>>>>>>>>>>>>>>>>>>>>>>>>>>>>>>>>>>>>>>>>>>>>>>>>>>>>>>>>>>>>>>>>>>>>>>>>>>>>>>>>>>>>>>>>>>>
Finished processing.
Number of putative recombinants: 46/7290 samples.
----------------------------------------------------------------------------------------------------
Execution time: 87 minutes
```

3.2 Correction for AF-distribution bias

The above initial filtering step resulted in 46 putative intra-host recombinants out of the 7,290 investigated co-infection samples. This drastic decrease in the number of eligible samples underlines the fact that our pipeline uses extremely strict filtering in order to separate true signs of recombination from sequencing noise.

To further refine these findings, we calculated the log-likelihood of observing the measured standardized alternate AFs in the sample given the putative breakpoint and also in a setting with no breakpoints. To determine these quantities, we first fitted bimodal distributions to the standardized alternate AF distribution of each mutually exclusive defining mutation of each variant in each variant combination. Given the $p(\overline{AF}_{s,g,A}, g, A, A{-}B)$ probability of observing $\overline{AF}_{s,g,A}$ standardized alternate AF at a defining mutation of variant A in a co-infection sample s with variant composition $A-B$ at genomic position g, the log-likelihood of measuring the given set of standardized AFs in a sample without a recombination breakpoint is:

$$\log \mathcal{L}_{nobp;s} = \sum_{g \in A} \log p(\overline{AF}_{s,g,A}, g, A, A-B) + \sum_{g \in B} \log p(\overline{AF}_{s,g,B}, g, B, A-B)$$

Assuming a recombination breakpoint at genomic position $g = b$, the log-likelihood becomes:

$$\log \mathcal{L}_{bp;s} = \sum_{g \in A; g \leq b} \log p(\overline{AF}_{s,g,A}, g, A, A-B) + \sum_{g \in A; g > b} \log p(\overline{AF}_{s,g,A}, g, A, A-B) + \\ + \sum_{g \in B; g \leq b} \log p(\overline{AF}_{s,g,B}, g, B, A-B) + \sum_{g \in B; g > b} \log p(\overline{AF}_{s,g,B}, g, B, A-B)$$

Note

Throughout this analysis, we assume that AF distributions at mutually exclusive defining mutations are independent.

Important

Restrictions on the value of g in the above formulas influence the set of defining mutations considered for the calculation of mean and standard deviation, which in turn affects the value of standardized AFs.

In the following steps, we collect those out of the 46 previously selected putative recombinants, for which the log-likelihood of a no-breakpoint model is lower than that of a breakpoint model.

```
Number of confirmed recombinants: 13/46 samples.
```

Fig. 5. shows the (non-standardized) AFs measured at defining positions for the remaining 13 samples, along with putative breakpoint positions. Before and after breakpoint means and standard deviations of relevant AFs are marked with horizontal dashed lines and shaded, semi-transparent regions. Odds-ratios of the breakpoint model vs. the no-breakpoint model and estimated ratios of the recombinant genome are also displayed.

Figure 5: Alternate AFs measured in mutually exclusive defining mutations of recombinant samples. The black vertical line shows the location of the putative breakpoint, dashed lines and shaded, semi-transparent regions mark the means and standard deviations of relevant AFs before and after the breakpoint. Odds ratios (OR) of the breakpoint model vs. the no-breakpoint model and the

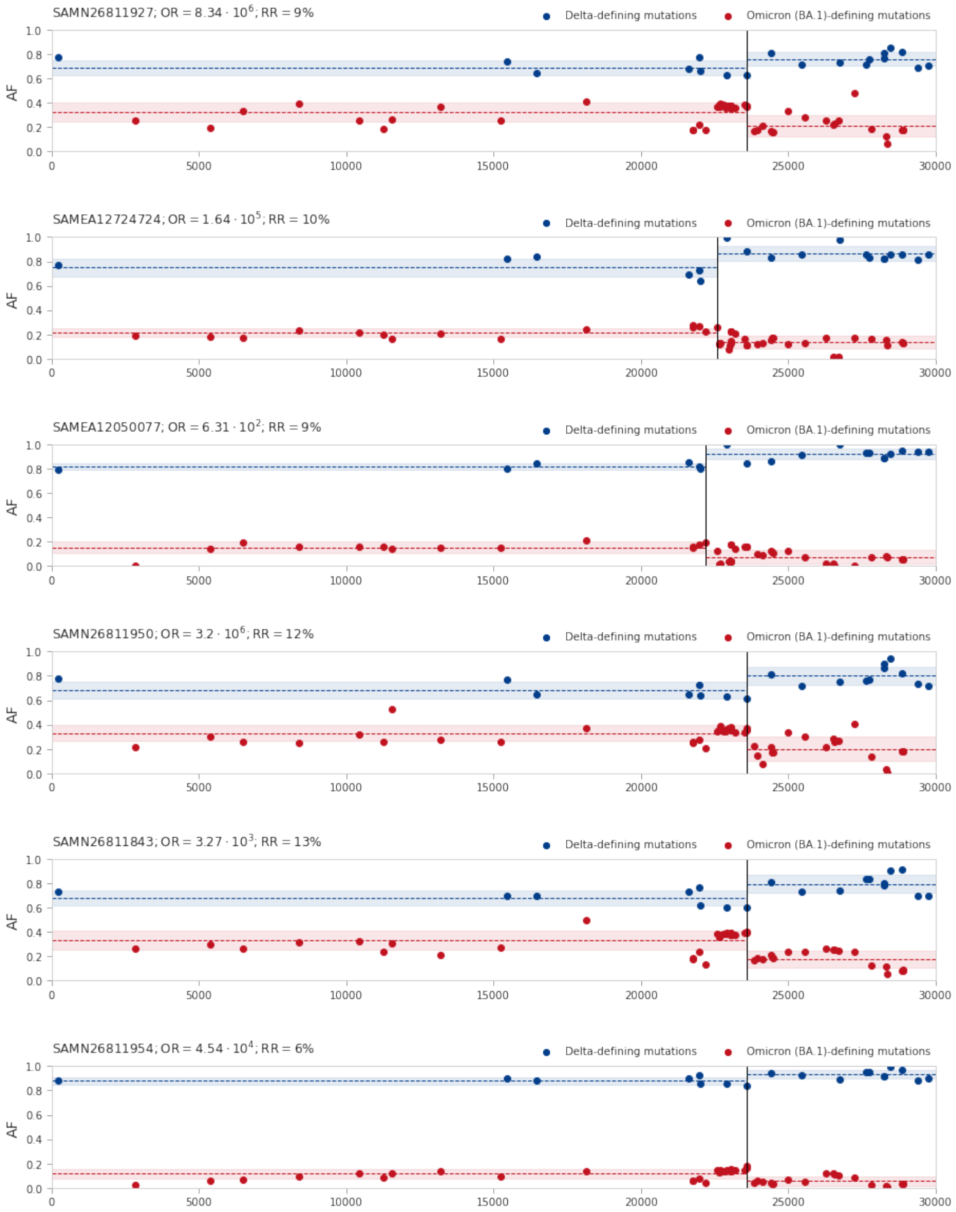

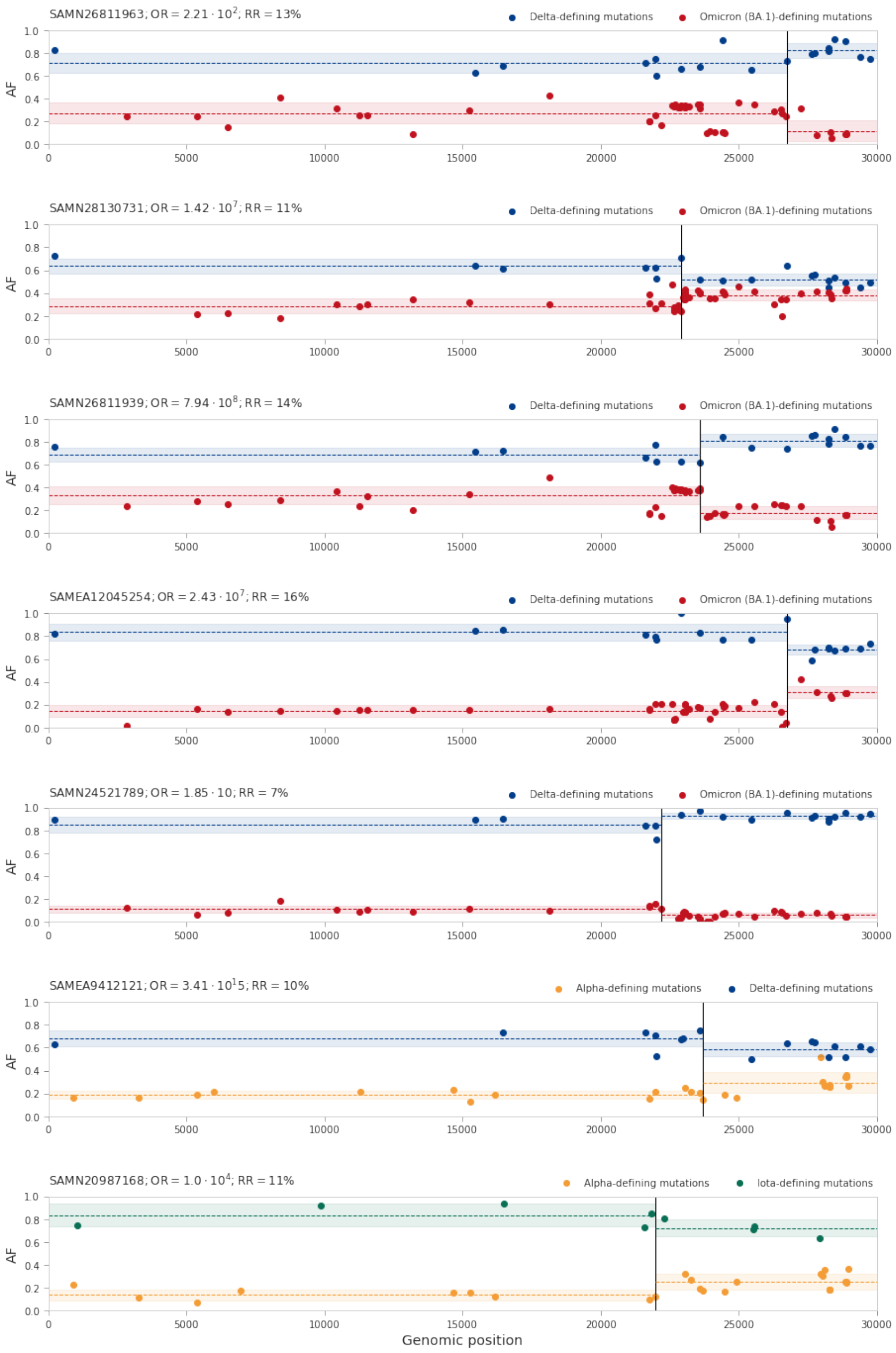

SAMN26811963; OR = 2.21 · 10^2; RR = 13%
Delta-defining mutations
Omicron (BA.1)-defining mutations
SAMN28130731; OR = 1.42 · 10^7; RR = 11%
Delta-defining mutations
Omicron (BA.1)-defining mutations
SAMN26811939; OR = 7.94 · 10^8; RR = 14%
Delta-defining mutations
Omicron (BA.1)-defining mutations
SAMEA12045254; OR = 2.43 · 10^7; RR = 16%
Delta-defining mutations
Omicron (BA.1)-defining mutations
SAMN24521789; OR = 1.85 · 10; RR = 7%
Delta-defining mutations
Omicron (BA.1)-defining mutations
SAMEA9412121; OR = 3.41 · 10^15; RR = 10%
Alpha-defining mutations
Delta-defining mutations
SAMN20987168; OR = 1.0 · 10^4; RR = 11%
Alpha-defining mutations
Iota-defining mutations
AF
0.0
0.2
0.4
0.6
0.8
1.0
0
5000
10000
15000
20000
25000
30000
Genomic position

Supplementary Method 3. - analysis pipeline

Traces of read-level recombination in coinfection samples

The online HTML version of the notebook contains codes for straightforward reproducibility and can be downloaded from https://github.com/csabaiBio/SARSCoV2-coinf/blob/main/pipelines/SuppFile3__read__pipeline.html. In this notebook, we analyse a subset of previously identified co-infection samples for the presence of short reads that overlap variant-defining mutations of both parental strains of the sample and carry both of these mutations simultaneously. We investigate how the genomic distribution of canonical lineage-specific mutations inherently affects the distribution of overlapping reads and thus the detectability of recombination breakpoints. We further examine reads carrying traces of recombination for recombination hotspots, traces of subgenomic RNA, comparability to AF-based analysis results and possible chimeric origin. As an introduction, we discuss the technical and theoretical challenges of intra-host recombinant detection.

Table of contents

1 Input and output files 1

2 Difficulties in detecting recombinants 2

3 Obtaining aligned sequencing data from ENA 4

4 Detection of overlapping and recombinant reads 4

5 Distribution of overlapping reads along the genome 5

6 Traces of recombination in overlapping reads 6

6.1 Distribution of recombination breakpoints along the genome . . . 6
6.2 Traces of recombination in artificial mixtures . . . 7
6.3 Common recombination breakpoint ranges . . . 7
6.4 Recombination in subgenomic RNA . . . 10
6.5 Recombinant reads in putative intra-host recombinant samples . . . 10

1 Input and output files

- Data files needed as input:
  - Additional data file 3 (https://github.com/csabaiBio/SARSCoV2-coinf/blob/main/data/datafile3.tar.gz)

    - Additional data file 5 (https://github.com/csabaiBio/SARSCoV2-coinf/blob/main/data/datafile5.tar.gz)

2 Difficulties in detecting recombinants

The detection of intra-host recombinant genomes in co-infection samples is hampered by multiple factors that together make it nigh impossible to reliably distinguish between true evidence of recombination and artefacts. Here we briefly summarize the main causes of this difficulty.

- **Subclonality:** Given that co-infection samples usually contain significant amounts of the two original parental strains, recombinant genomes comprise only a small portion of the sequenced viral population. Thus any attempt at identifying recombinants must reckon with decreased coverage and consequently a limited amount of available data.
- **PCR artefacts:** PCR amplification is the standard method for the generation of sufficient genetic material prior to sequencing. Most of the sequencing data for SARS-CoV-2 has been produced by a pipeline that incorporates PCR amplification in its initial steps. Admittedly, there are a few experimental setups in which metagenomic sequencing was employed, which eliminates various problematic issues introduced by PCR-based methods, however, the abundance of SARS-CoV-2 genomes in these samples is usually too low to draw meaningful conclusions.
    - **Systematic bias in alternate allele frequency distribution:** It has been previously shown that alternate allele frequencies measured at defining mutations in artificial samples of mixed variants do not correctly reflect the original mixture proportions in the sample (Bal et al., 2022). Additionally, based on our observations, a systematic bias can be identified in the alternate allele frequency distribution of defining mutations in co-infection samples of specific variant combinations (see Supplementary file 2.). We hypothesize that this effect might be due to the preferential attachment of primers to one of the parental viral strains that carries a set of favourable or lacks a set of disadvantageous mutations. As a result of this, the presence of intra-host recombinant genomes cannot be reliably detected by subtle shifts of alternate allele frequencies along the genome.
    - **Chimeras:** It has been known for decades that during PCR amplification, PCR-mediated recombination or "chimera formation" systematically occurs (Brakenhoff et al., 1991), generating artificial sequences that are essentially no different from true recombinants. It is virtually impossible to dependably distinguish between these, thus one has to assume that chimera formation is relatively rare, while viral recombination is well-documented in laboratory settings, hence also expected to occur in co-infection samples.
- **Subgenomic RNA:** Besides virus genome length RNA, diagnostic samples of SARS-CoV-2 have been shown to carry leader sequence-containing subgenomic RNAs (sgRNAs) as well (Kim et al., 2020, Alexandersen et al., 2020). Recombination occurring in the sgRNAs has no effect on viral evolution and cannot be passed to future viral populations, thus its presence is less relevant than recombination of the genomic RNA. Short reads showing signs of recombination that contain the so-called leader sequence and/or were soft-clipped during alignment can be relatively confidently categorized as originating from sgRNA. However, due to short read lengths and fairly long sequences of the transcriptome, a short read might still be sgRNA-derived, even without the presence of the leader sequence.
- **The low number of defining mutations:** Given that, disregarding the relatively low number of defining mutations, parental strains in SARS-CoV-2 co-infection samples are highly similar, recombination events might go completely undetected. The identification of recombination breakpoints

is limited to the genomic ranges between defining mutations, thus the uncertainty in their location is extremely high.

- **Uneven distribution of defining mutations:** Defining mutations are unevenly distributed across genomic positions, a disproportionately high amount (considering gene lengths) of them are located on genes S and N (Fig. 1), making it very difficult to detect recombination breakpoints occurring in other, less frequently mutated regions of the genome.
- **Short read lengths:** Direct evidence of recombination events can come from short reads that simultaneously contain the defining mutations of both parental strains in a co-infection sample. This approach, however, is limited by the relatively short read lengths (100-200 bp) in sequencing data generated by Illumina platforms, as only those defining mutation pairs are overlapped by the same reads that are located close enough on the genome. The recent advances in Nanopore sequencing technologies might provide a solution for this problem, as they usually generate reads ranging from 10 to 100 kbp in length.

In the following analysis, we collect reads overlapping defining mutations of multiple variant stains and show how their detectability is largely influenced by the presence and distribution of variant-defining mutations. We also examine them for traces of recombination and discuss the resulting distribution of recombination breakpoints and how it is biased by the factors described above.

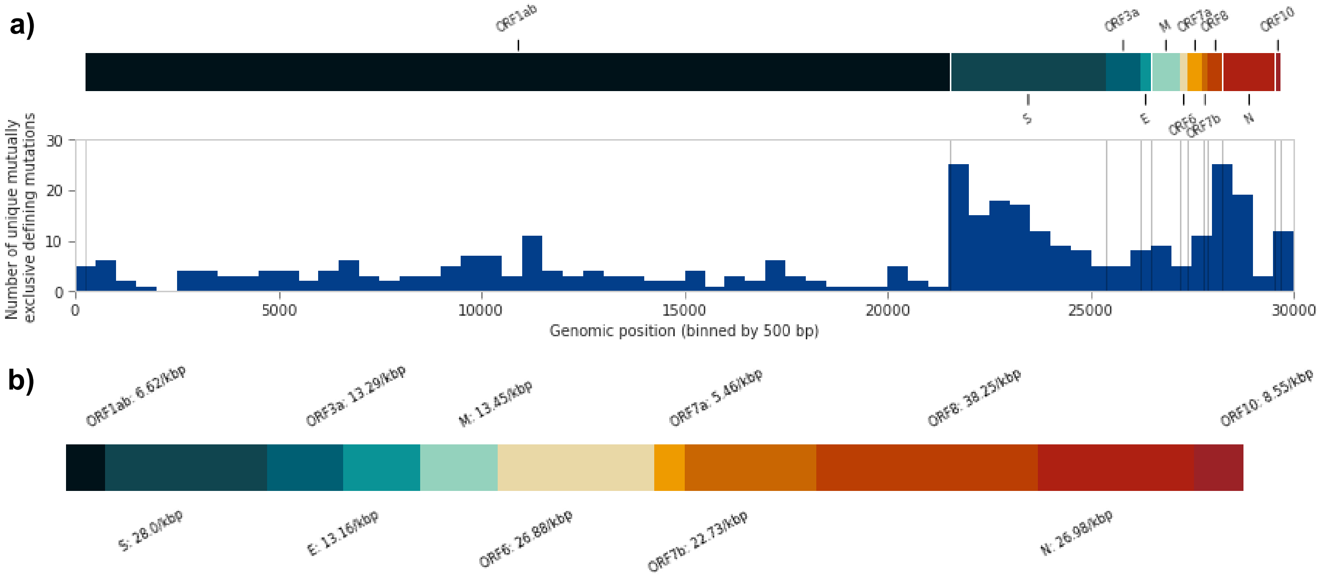

Figure 1: Location and density of mutually exclusive defining mutations in the SARS-CoV-2 genome. a) Distribution of mutually exclusive defining mutations along the SARS-CoV-2 genome, binned by 500 bp. All mutations are included that are considered to be mutually exclusive defining mutations of a variant in any of the variant combinations of the 7,413 co-infection samples. Each specific mutation has been counted once to create this figure. The top panel shows the locations of specific genes along the genome. Vertical lines on the bottom panel indicate gene boundaries. b) Mutually exclusive defining mutation density in various genes. Mutations not overlapping any of the canonical genes have been discarded for this figure. The widths of coloured regions are proportional to the density of defining mutations in the genes.

3 Obtaining aligned sequencing data from ENA

The analyses in this notebook use raw, aligned sequencing data (BAM files) to identify reads that carry defining mutations of multiple variants. In order to perform these investigations, the database of the European Nucleotide Archive (ENA) was queried for the location of BAM files of a list of previously selected co-infection samples and data was then locally downloaded for downstream analysis.

> **Note**
>
> The original set of previously detected 7,413 co-infection samples was first limited to those 6,999 that contained traces of exactly two variants. (I.e. samples identified as the mixtures of three or more variant strains were discarded.) Then the resulting set of samples was further downsampled to a set of 100 samples for this analysis to decrease computation time and simultaneously preserve the prevalence of specific variant combinations. The 13 co-infection samples identified as putative intra-host recombinants based on alternate allele frequency (AF) shifts along their genome (see Supplementary File 2) were added to this list, along with 5 artificial mixture samples of study PRJNA827817, resulting in altogether 118 samples for this analysis.

4 Detection of overlapping and recombinant reads

We principally used the `mpileup` command of the `samtools` software to identify reads that overlapped at least one mutually exclusive defining mutation of both the comprising variant strains and carried the alternate allele in both of these genomic positions. The following pipeline was implemented for the analysis:

1. Get the pileup of the BAM file for all genomic positions in the sample where a mutually exclusive mutation of one of the comprising variant strains is present.
   - filter for only these positions (`-l position_list.txt`)
   - disable base alignment quality (BAQ) computation (`-B`)
   - filter for both base (`-Q 30`) and mapping quality (`-q 30`)
   - disable sequencing depth cutoff (`-d 0`)
   - also output read IDs for each detected base (`--output-QNAME`)
2. Find all possible pairs of genomic positions (from the above list), where one comprising strain has a mutually exclusive defining mutation in one of the positions and the other strain has one in the other position. Query the pileup for these pairs of genomic positions.
3. Determine the number of reads overlapping both positions, based on the read IDs provided in the pileup.
4. Categorize each overlapping read as one of the following:
   - the read carries the mutually exclusive defining mutation of one variant strain,
   - the read carries the mutually exclusive defining mutation of the other variant strain,
   - the read carries the mutually exclusive defining mutation of *both* variant strains (i.e. supports recombination),
   - the read carries neither mutually exclusive defining mutations.
5. Calculate the numbers of reads belonging to each group for all relevant mutation pairs in the sample and save the results.

5 Distribution of overlapping reads along the genome

As previously discussed, the number of reads that overlap mutually exclusive defining mutations of both comprising variants is largely influenced by the density of these mutations along the genome. Hereby we define the location of an overlapping read as the midpoint of the defining mutation pair it overlaps. Fig. 2a depicts the distribution of overlapping read locations with a 500 bp binning with a logarithmic vertical scale. Overlapping reads were counted together for all 118 analysed samples. The left panel of Fig. 2b shows the correlation between the number of overlapping reads at a given genomic range and the number of mutually exclusive defining mutations that fall into that same range. The right panel of Fig. 2b displays the same results for the density of mutually exclusive defining mutations and overlapping reads in specific genes.

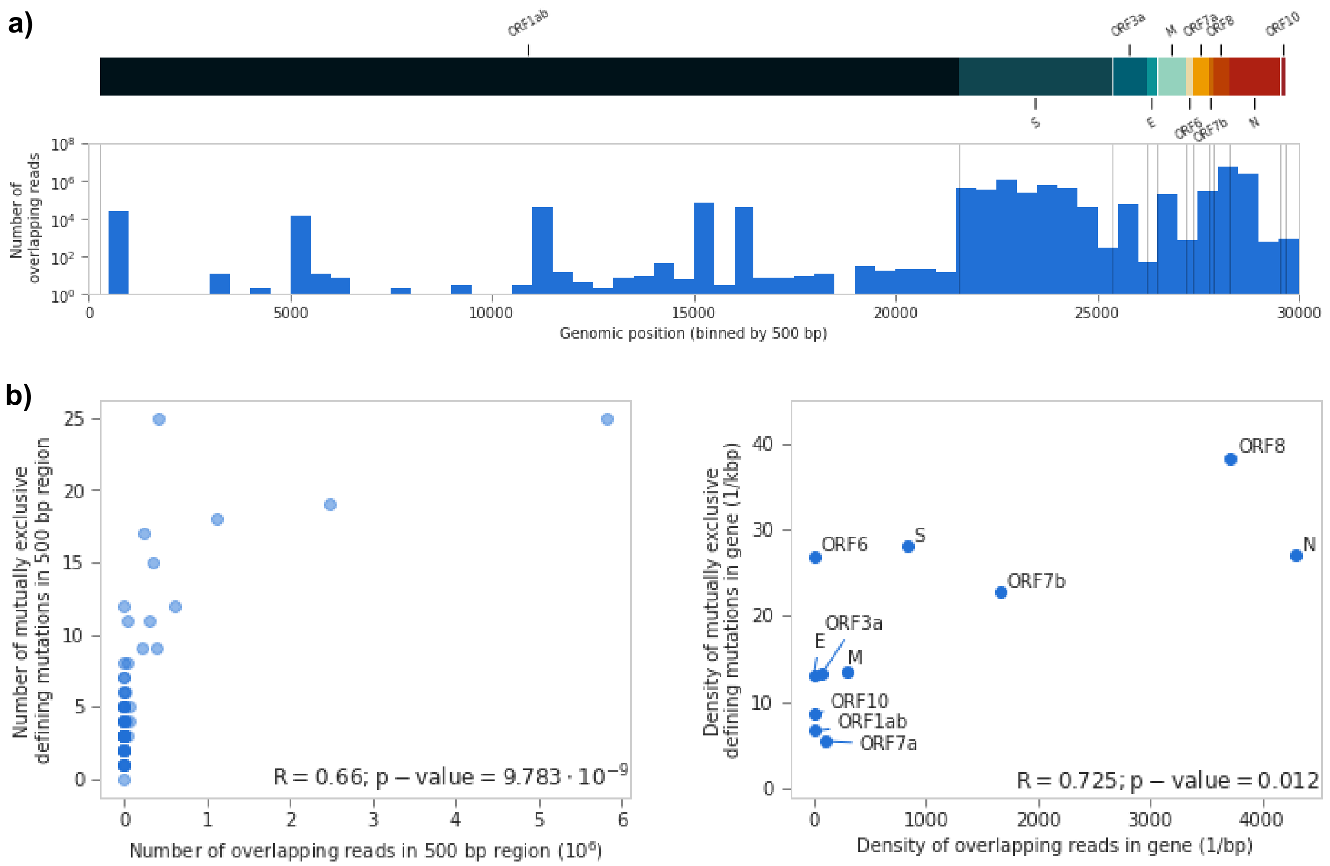

Figure 2: Distribution of the locations of reads overlapping mutually exclusive defining mutations of both comprising variants. a) Distribution of overlapping read locations along the SARS-CoV-2 genome, binned by 500 bp. All overlapping reads of the 118 analysed samples were considered for this figure. Overlapping read location is defined as the midpoint of mutually exclusive defining mutations it overlaps. The top panel shows the locations of specific genes along the genome. Vertical lines on the bottom panel indicate gene boundaries. Note that the scale of the vertical axis is logarithmic. b) Left: Correlation of the number of mutually exclusive defining mutations and the number of overlapping reads in 500 bp genomic regions. Right: Correlation of the density of mutually exclusive defining mutations and the density of overlapping reads in specific genes.

6 Traces of recombination in overlapping reads

6.1 Distribution of recombination breakpoints along the genome

We define the location of a recombination breakpoint as the midpoint of the recombination range. The recombination range is the region of the genome bracketed by two mutually exclusive defining mutations (one of each parental strain) that are overlapped by at least one read in which both mutations are present. The distribution of recombination breakpoints is displayed in Fig. 3a for all 118 analysed co-infection samples. In samples where multiple reads support a single breakpoint, the breakpoint is counted only once.

It has recently been shown that recombination breakpoints occur non-uniformly across the viral genome (Turakhia et al., 2022). However, the computation approach (named "RIPPLES") providing this finding takes consensus sequences as its input. In order to compare its results with our read-level data, we downloaded Supplementary Data 1 of the above manuscript and calculated the correlation between the number of breakpoints detected by RIPPLES and the number of breakpoints supported by overlapping reads with a 500 bp binning along the genome. The results of this comparison are shown in Fig. 3b.

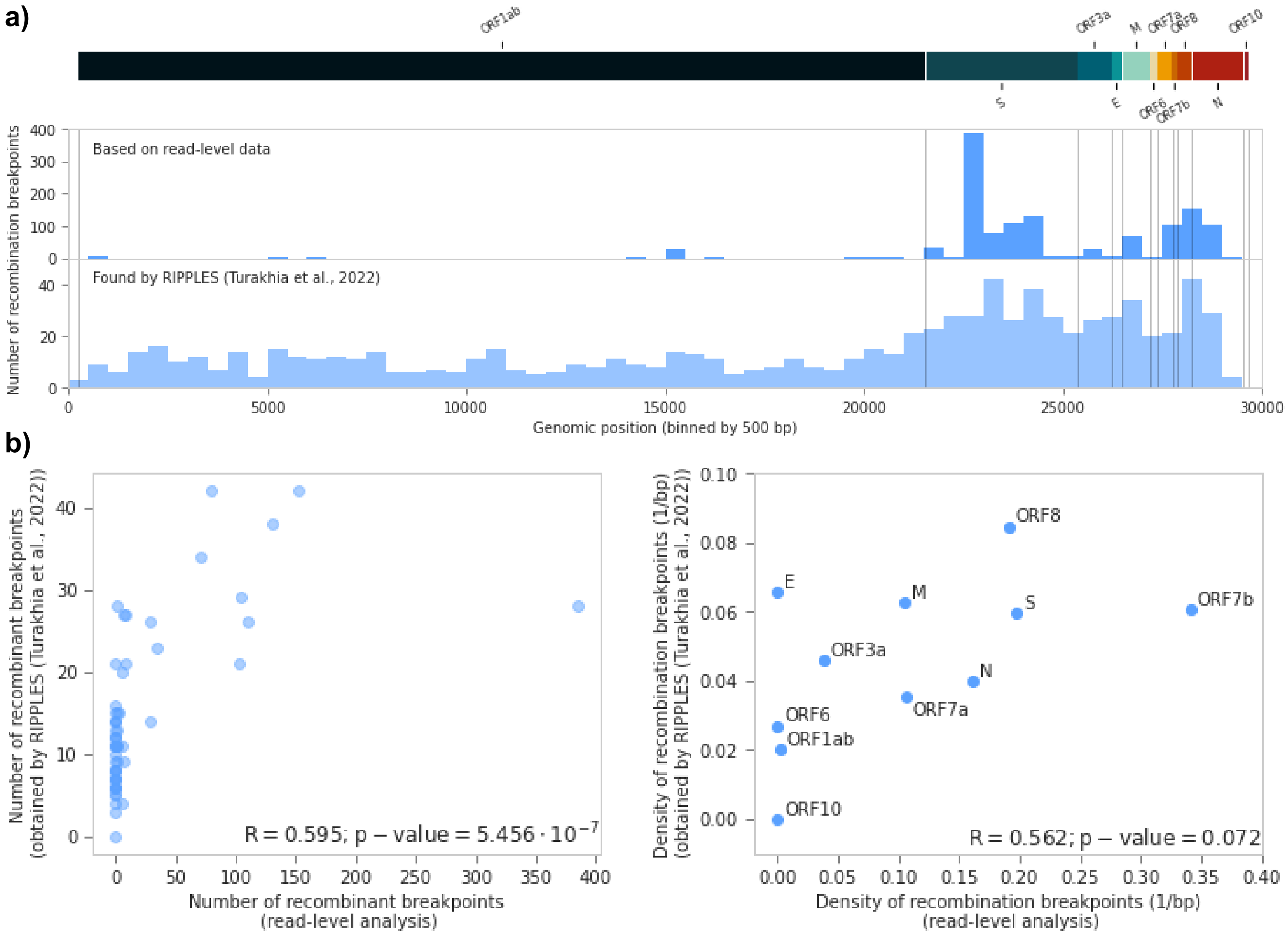

Figure 3: Distribution of recombination breakpoints along the genome. a) Upper panel: Number of recombination breakpoints in 500 bp-wide regions of the SARS-CoV-2 genome based on overlapping reads containing multiple defining mutations of the parental strains. Lower panel: Distribution of recombination breakpoints along the genome with a 500 bp binning found by RIPPLES, based on Turakhia et al., 2022. b) Left: Relationship between the number of recombination breakpoints indicated by overlapping reads and the number of breakpoints identified by Thurakia et al. 2022 (with the RIPPLES software) in consensus sequences. The genome was binned by 500 bp regions for this analysis. Right: Correlation of the density of recombination breakpoints indicated by overlapping reads and the density of breakpoints identified by Thurakia et al. 2022 in specific genes.

The above results indicate that the findings of RIPPLES and the results of the read-level analysis are moderately correlated, thus suggesting that both are largely influenced by the inherent distribution of defining mutations along the genome.

6.2 Traces of recombination in artificial mixtures

In theory, artificial mixtures of purified RNA from different viral strains could serve as reliable controls for recombinant detection, as no recombination is expected to occur once viral replication has been terminated. In our set of samples selected for read-level analysis, 7 samples of Bal et al., 2022 (study ID PRJNA817870) and 5 samples of Sovic et al., 2022 (study ID PRJNA827817) are in fact artificial mixtures, thus it is of interest to examine the distribution of recombinant reads along their genomes.

Artificial mixture samples do contain a substantial number of recombinant reads, but the prevalence of genomic positions overlapped by a recombinant read ratio of more than 0.1 (vertical black lines) is much lower than in true co-infection samples. This result suggests that breakpoints supported by no more than 10% of the overlapping reads might be considered artefacts due to chimera formation during PCR.

This is further supported by the fact that when calculating the ratio of duplicate reads among recombinant reads in positions with a recombinant read ratio of lower vs. higher than 10%, genomic positions in which recombination was supported by less than 10% of overlapping reads show very high fractions of duplicates, indicating possible evidence of PCR artefacts.

6.3 Common recombination breakpoint ranges

To check whether any recombination breakpoints systematically occur in our investigated samples, we plotted the ratio of samples in which sufficient evidence of a breakpoint (supported by at least 10 reads and a recombination read ratio of more than 0.1) can be uncovered for the given genomic position. Co-infection samples of the 4 most common variant combinations were considered for this figure. Artificial samples were discarded during this analysis.

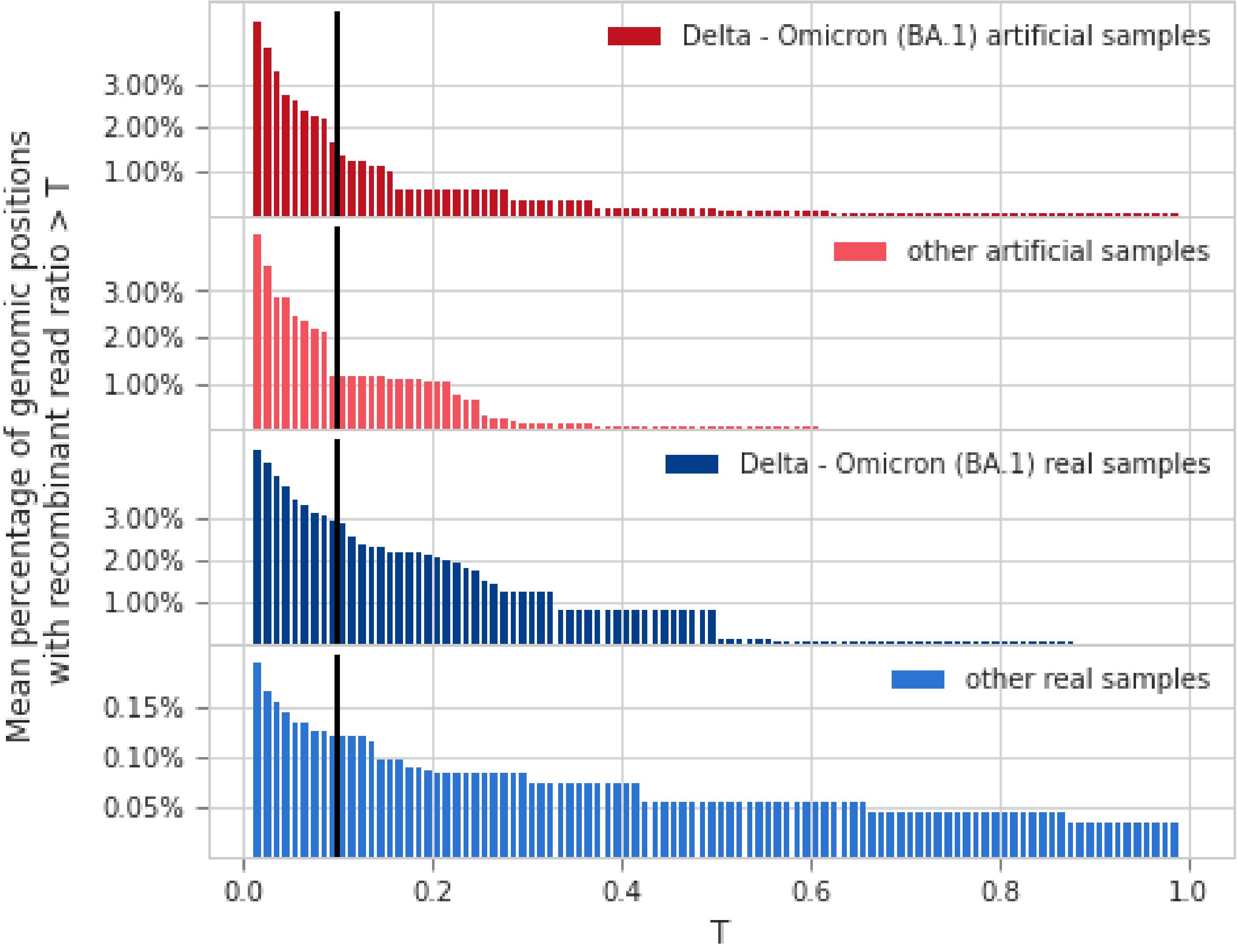

Figure 4: The average percentage of genomic positions (per sample) for which the ratio of recombinant reads out of all overlapping ones reaches T. Genomic positions with exactly zero recombinant reads are not shown. Samples were categorized into groups of Delta − Omicron (BA.1) artificial/real and non-Delta − Omicron (BA.1) artificial/real samples. The vertical black line indicates T = 0.1.

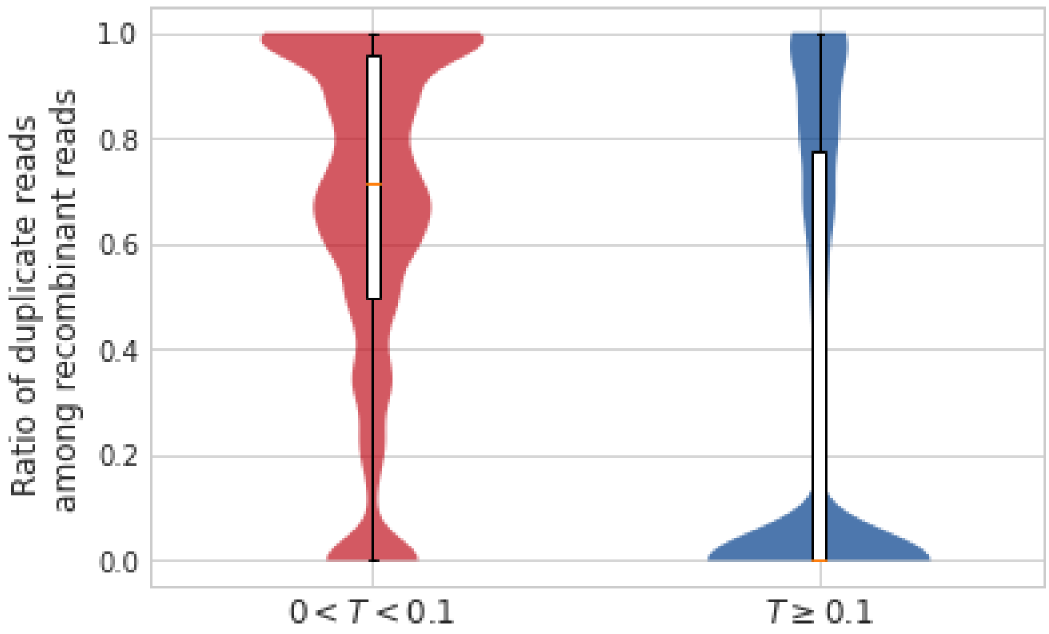

Figure 5: The ratio of duplicate reads among recombinant reads in genomic positions with a recombinant read ratio less than vs. at least 10%. Genomic positions with no recombinant reads were excluded from this figure.

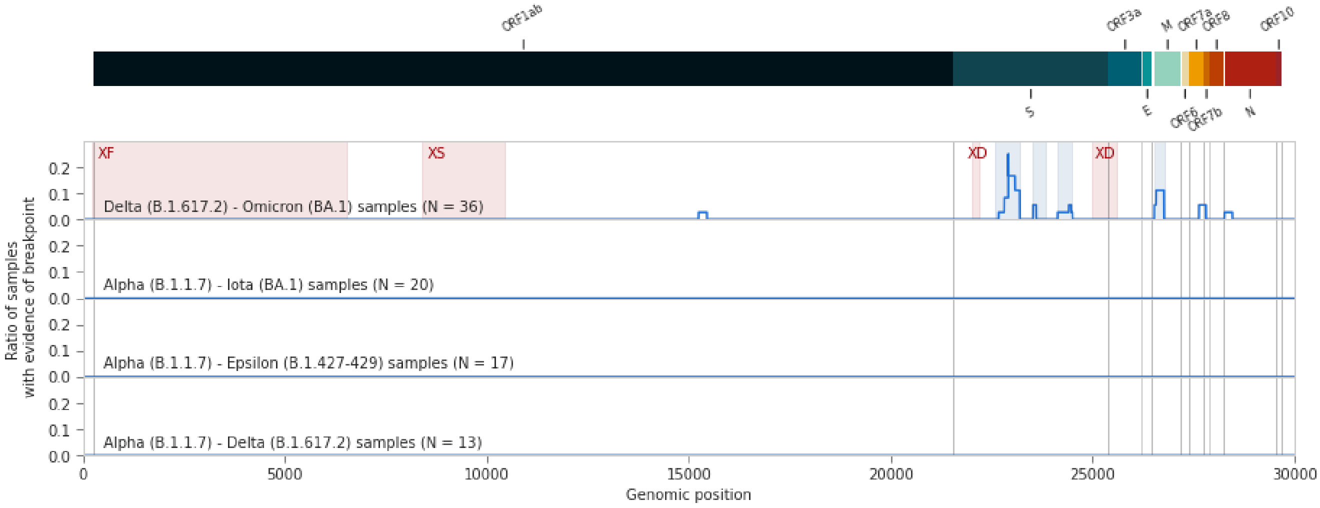

Figure 6: The ratio of various co-infection samples with sufficient evidence of a recombination breakpoint in different genomic positions. For this analysis, co-infection samples were categorized based on their variant composition. Only those variant compositions are shown for which at least 10 samples were available in the raw read analysis pipeline. For each genomic position, the number of samples was calculated in which the given position is part of a recombination breakpoint range based on the evidence of at least 10 short reads and a recombinant read ratio of 0.1 or larger. For each sample, each genomic position was counted a single time only. Regions shaded with light red are recombination breakpoint ranges of the three Pangolin lineages (XF, XS, and XD) in which Delta – Omicron recombination occurs. Areas shaded with light blue indicate intragenic hotspots. Artificial samples were not included in this analysis.

Many of the genomic ranges indicated as putative recombination breakpoint ranges in multiple samples coincide with gene boundaries. Additional intragenic hotspots were regions 22578-23202, 23525-23854, and 24130-24503 in gene S and 26530-26767 in gene M in co-infection samples of Delta – Omicron (BA.1) variants (shaded with light blue above). The majority of recombination hotspots detected from short reads do not correspond to regions of recombination identified from clonal recombinants of the GISAID database (samples assigned to Pango lineages XF, XS and XD, shaded with light red above; for more details, see Supplementary Figure 3).

6.4 Recombination in subgenomic RNA

In order to check if reads carrying traces of recombination originate from subgenomic RNA sequences, we first collected read IDs that were detected as recombinants for each sample, for each relevant pair of mutually exclusive defining mutations and then queried the original BAM files for their details. We checked whether these reads contained the nucleotides of the common 5'-leader sequence attached to sgRNAs during translation and if they were soft-clipped during alignment. If any of these conditions were met, the given read was considered to show signs of sgRNA-origin.

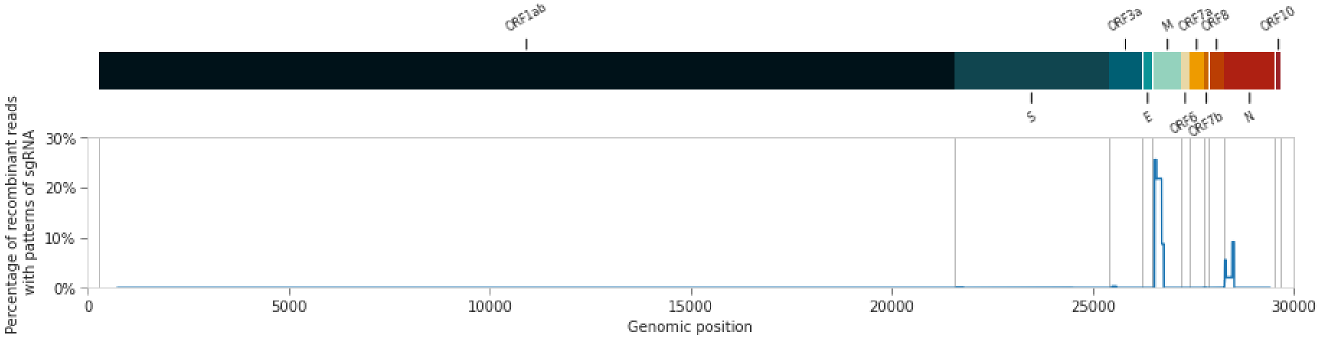

Figure 7: The percentage of recombinant reads carrying signs of originating from sgRNA along the genome. Artificial samples were not considered for this analysis.

Leader sequences and soft-clipping in recombinant reads were almost exclusively present when the reads overlapped gene boundaries (S/ORF3a, E/M and ORF8/N). This is in line with our previous knowledge about sgRNA formation and it also suggests that recombinant reads overlapping intragenic regions are unlikely to originate from sgRNA and are rather products of recombination occurring on the genomic RNA.

6.5 Recombinant reads in putative intra-host recombinant samples

Based on measured AF shifts along the genome, 13 samples have been selected as showing putative signs of recombination (see Supplementary File 2). (Note, that 6 of these were artificial mixtures of study PRJNA817870 from Bal et al., 2022.)

The figures below illustrate the distribution of recombinant reads in these 13 samples along with the putative recombination breakpoint that was determined from the location of the AF shift of defining mutations (Supplementary File 2).

None of the putative breakpoints identified from AF analysis (Supplementary File 2) could be verified from read-level data with a recombinant read ratio of 0.1 or larger (either for artificial or real samples). In many cases, the number of reads overlapping the putative breakpoint was ab ovo very low.

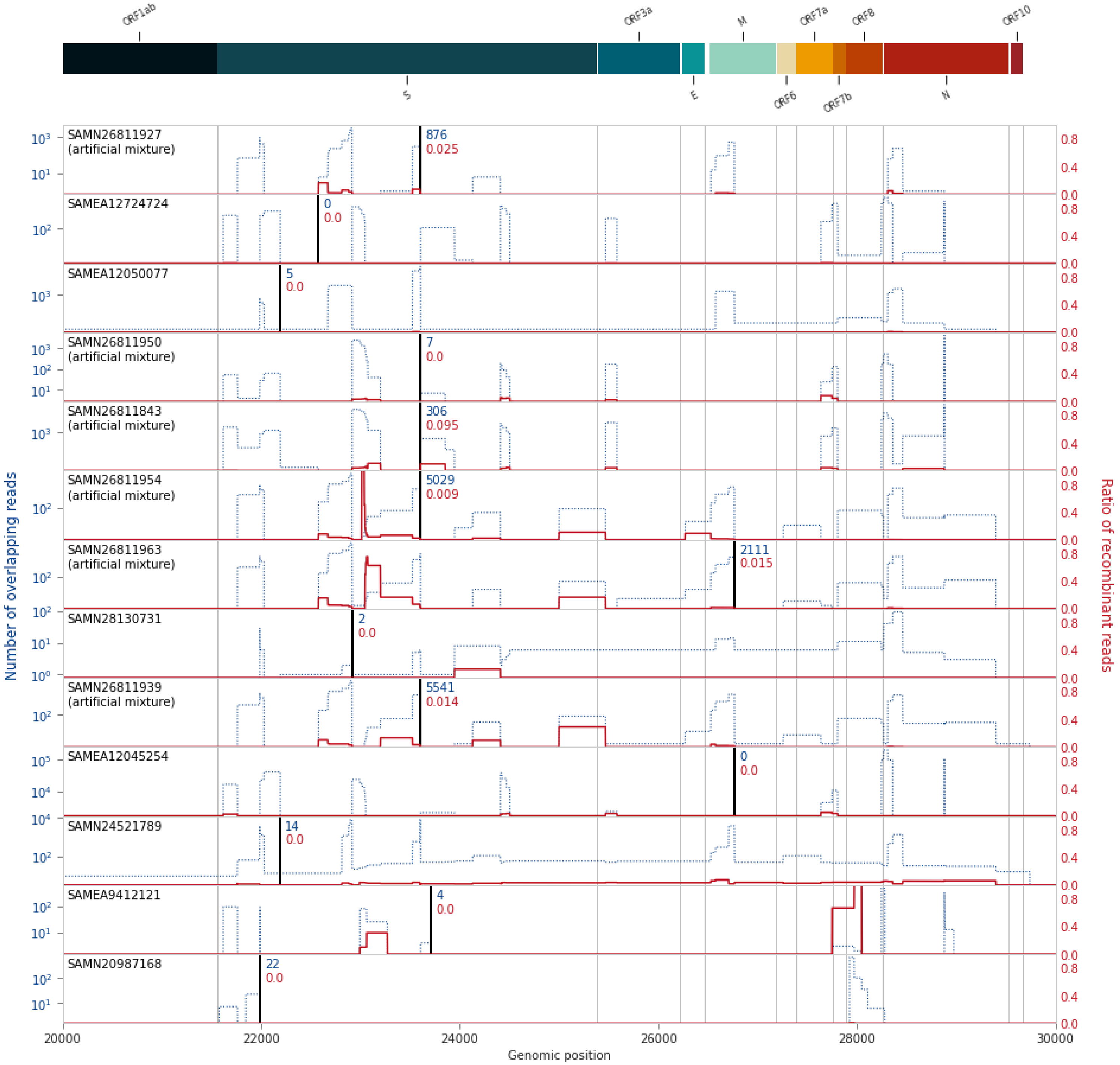

Figure 8: The number of overlapping reads (dotted blue lines) and the ratio of ones showing signs of recombination among them (solid red lines) for samples identified as putative intra-host recombinants from shifts in AF distribution. The figure is limited to the 20,000-30,000 genomic position range, as putative recombination breakpoints (shown with black vertical lines) were also confined to this region. Blue and red numbers right after the breakpoints indicate the number of reads overlapping the breakpoint and the ratio of ones carrying signs of recombination among these.

Supplementary References

1. Khare, S. *et al.* GISAID's Role in Pandemic Response. *China CDC Wkly* **3**, 1049–1051 (2021).
2. O'Toole, Á. *et al.* Assignment of epidemiological lineages in an emerging pandemic using the pangolin tool. *Virus Evol* **7**, (2021).
3. Versatile Emerging infectious disease Observatory (VEO). https://www.veo-europe.eu/ (2023).
4. VEO-Covid Sequence Analysis Workflow. Illumina. https://github.com/enasequence/covid-sequence-analysis-workflow/blob/master/illumina/illumina.nf (2023).
5. VEO-Covid Sequence Analysis Workflow. Nanopore. https://github.com/enasequence/covid-sequence-analysis-workflow/blob/master/nanopore/nanopore.nf (2023).
6. Harrison, P. W. *et al.* The COVID-19 Data Portal: accelerating SARS-CoV-2 and COVID-19 research through rapid open access data sharing. *Nucleic Acids Res* **49**, W619–W623 (2021).
7. Rahman, N. *et al.* Mobilisation and analyses of publicly available SARS-CoV-2 data for pandemic responses. *bioRxiv* 2023.04.19.537514 (2023) doi:10.1101/2023.04.19.537514.
8. Valieris, R. *et al.* A mixture model for determining SARS-Cov-2 variant composition in pooled samples. *Bioinformatics* (2022) doi:10.1093/bioinformatics/btac047.
