# Supplementary material for: Systematic detection of co-infection and intra-host recombination in more than 2 million global SARS-CoV-2 samples
Source: Nat Commun. 2024 Jan 15;15:517. doi: 10.1038/s41467-023-43391-z (PMC10789779; doi:10.1038/s41467-023-43391-z)
Supplement: Supplementary file 3 — Description of Additional Supplementary Files [file 41467_2023_43391_MOESM3_ESM.pdf]

Description of Additional Supplementary Files

File Name: Supplementary Data 1
Description: Co-infection prevalence in different studies. Studies are listed in decreasing order based on the total number of their good-quality samples. Studies highlighted in grey had co-infection prevalence larger than 20%.

File Name: Supplementary Data 2
Description: List of unique variant-defining mutations used in the study. Samples presenting a ratio of at least 0.5 of unique defining mutations for at least two different variants were retained for further analysis. Prevalences indicate the percentage of GISAID samples assigned to the specific variant that had the given mutation.

File Name: Supplementary Data 3
Description: Number of unique variant-defining mutations used in the study for each investigated variant.

File Name: Supplementary Data 4
Description: Mutually exclusive defining mutations of specific variant combinations, defined as mutations present in at least 80% of GISAID samples assigned to the given variant, while simultaneously present in less than 10% of the samples assigned to any other variant(s) of the variant combination. (Variant combinations for which no more than 10 putative co-infection samples were detected based on unique defining mutations alone (Supplementary Data 2 and 3) are not listed.)
