# Supplementary material for: Systematic detection of co-infection and intra-host recombination in more than 2 million global SARS-CoV-2 samples
Source: Nat Commun. 2024 Jan 15;15:517. doi: 10.1038/s41467-023-43391-z (PMC10789779; doi:10.1038/s41467-023-43391-z)
Supplement: Supplementary file 8 — Reporting Summary [file 41467_2023_43391_MOESM8_ESM.pdf]

nature portfolio

Corresponding author(s): Anna Medgyes-Horváth

Last updated by author(s): Oct 31, 2023

Reporting Summary

Nature Portfolio wishes to improve the reproducibility of the work that we publish. This form provides structure for consistency and transparency in reporting. For further information on Nature Portfolio policies, see our Editorial Policies and the Editorial Policy Checklist.

Statistics

For all statistical analyses, confirm that the following items are present in the figure legend, table legend, main text, or Methods section.

n/a	Confirmed	
☐	☒	The exact sample size (*n*) for each experimental group/condition, given as a discrete number and unit of measurement
☒	☐	A statement on whether measurements were taken from distinct samples or whether the same sample was measured repeatedly
☐	☒	The statistical test(s) used AND whether they are one- or two-sided<br>*Only common tests should be described solely by name; describe more complex techniques in the Methods section.*
☒	☐	A description of all covariates tested
☒	☐	A description of any assumptions or corrections, such as tests of normality and adjustment for multiple comparisons
☐	☒	A full description of the statistical parameters including central tendency (e.g. means) or other basic estimates (e.g. regression coefficient) AND variation (e.g. standard deviation) or associated estimates of uncertainty (e.g. confidence intervals)
☐	☒	For null hypothesis testing, the test statistic (e.g. *F*, *t*, *r*) with confidence intervals, effect sizes, degrees of freedom and *P* value noted<br>*Give P values as exact values whenever suitable.*
☒	☐	For Bayesian analysis, information on the choice of priors and Markov chain Monte Carlo settings
☒	☐	For hierarchical and complex designs, identification of the appropriate level for tests and full reporting of outcomes
☐	☒	Estimates of effect sizes (e.g. Cohen's *d*, Pearson's *r*), indicating how they were calculated

Our web collection on statistics for biologists contains articles on many of the points above.

Software and code

Policy information about availability of computer code

Data collection	No software was used for data collection.
Data analysis	Analysis pipelines are available in the SARSCoV2-coinf GitHub repository at github.com/csabaiBio/SARSCoV2-coinf (DOI: 10.5281/zenodo.10057335).<br>The CoVEO database uses PostgreSQL version 11.12. Analyses were performed in python version 3.8.8 with the help of python modules psycopg2 (v2.9.5), pandas (v1.5.3), matplotlib (v3.3.4), numpy (v1.21.6), IPython (v7.22.0), tabulate (v0.9.0), scipy (v1.7.3) and re (v2.2.1).<br>Aligned BAM files were manipulated with samtools63 version 1.16. Further visualisations were created in R version 4.2.1 with R packages dplyr (v1.0.10), tidyr (v1.2.1), ggplot2 (v3.3.6), ISOweek (v0.6-2), stringr (v1.4.1), scales (v1.1.0), jsonlite (v1.8.2), ggpubr (v0.2.5), ggrepel (v0.9.1) and ComplexHeatmap (v2.12.1).

For manuscripts utilizing custom algorithms or software that are central to the research but not yet described in published literature, software must be made available to editors and reviewers. We strongly encourage code deposition in a community repository (e.g. GitHub). See the Nature Portfolio guidelines for submitting code & software for further information.

Data

Policy information about availability of data

All manuscripts must include a data availability statement. This statement should provide the following information, where applicable:

- Accession codes, unique identifiers, or web links for publicly available datasets
- A description of any restrictions on data availability
- For clinical datasets or third party data, please ensure that the statement adheres to our policy

The analysed VCF and BAM files created with the unified pipelines are accessible under the EBI-ENA umbrella project PRJEB45555 [https://www.ebi.ac.uk/ena/browser/view/PRJEB45555].
GISAID sequences used in this study are accessible [https://doi.org/10.55876/gis8.231020pu].
Datasets created and further processed in the current study, along with all supplementary material are available in the SARSCoV2-coinf GitHub repository at github.com/csabaiBio/SARSCoV2-coinf (DOI: 10.5281/zenodo.10057335).

Research involving human participants, their data, or biological material

Policy information about studies with human participants or human data. See also policy information about sex, gender (identity/presentation), and sexual orientation and race, ethnicity and racism.

Reporting on sex and gender	N/A
Reporting on race, ethnicity, or other socially relevant groupings	N/A
Population characteristics	N/A
Recruitment	N/A
Ethics oversight	N/A

Note that full information on the approval of the study protocol must also be provided in the manuscript.

Field-specific reporting

Please select the one below that is the best fit for your research. If you are not sure, read the appropriate sections before making your selection.

☒ Life sciences ☐ Behavioural & social sciences ☐ Ecological, evolutionary & environmental sciences

For a reference copy of the document with all sections, see nature.com/documents/nr-reporting-summary-flat.pdf

Life sciences study design

All studies must disclose on these points even when the disclosure is negative.

Sample size	Sample size calculation was not performed, as all 3,093,454 publicly available SARS-CoV-2 samples with a processed list of mutations deposited to the European COVID-19 Data Portal (https://www.covid19dataportal.org/) was included in the initial analysis.
Data exclusions	Samples were filtered for sequencing quality and only those were kept that had a total base count of at least 100,000 and had a sequencing depth of less than 10 in more than 10% of the 29,903 genomic positions of the reference genome. This filtering step resulted in 2,172,927 remaining samples collected in the period from the 30th of December 2019 to the 30th of June 2022.
Replication	No replication was performed as the study utilizes worldwide data in an attempt to simultenously analyse as many samples as possible, so the complete reproduction of the dataset was unfeasible. However, we re-analysed samples previously considered for co-infection detection in various other studies and obtained consistent, robust results comparable to previous findings.
Randomization	Randomization was not relevant to our study, as samples were not assigned to experimental groups and all available data was analysed.
Blinding	Blinding was not relevant to our study, as group allocations were not performed.

Reporting for specific materials, systems and methods

We require information from authors about some types of materials, experimental systems and methods used in many studies. Here, indicate whether each material, system or method listed is relevant to your study. If you are not sure if a list item applies to your research, read the appropriate section before selecting a response.

Materials & experimental systems

n/a	Involved in the study
☒	☐ Antibodies
☒	☐ Eukaryotic cell lines
☒	☐ Palaeontology and archaeology
☒	☐ Animals and other organisms
☒	☐ Clinical data
☒	☐ Dual use research of concern
☒	☐ Plants

Methods

n/a	Involved in the study
☒	☐ ChIP-seq
☒	☐ Flow cytometry
☒	☐ MRI-based neuroimaging
